# *Vitex* Genus as a Source of Antimicrobial Agents

**DOI:** 10.3390/plants13030401

**Published:** 2024-01-29

**Authors:** Zohorul Islam, Gonçalo I. Caldeira, Manuela Caniça, Nurul Islam, Olga Silva

**Affiliations:** 1 Research Institute for Medicines (iMed.ULisboa), Faculty of Pharmacy, University of Lisbon, 1649-003 Lisbon, Portugal; zislam@edu.ulisboa.pt (Z.I.); goncalo.caldeira@edu.ulisboa.pt (G.I.C.); 2 National Reference for Laboratory of Antibiotic Research and Healthcare-Associated Infections, Department of Infectious Diseases, National Institute of Health Dr. Ricardo Jorge, 1649-016 Lisbon, Portugal; manuela.canica@insa.min-saude.pt; 3 Department of Zoology, Faculty of Biological Sciences, University of Rajshahi, Rajshahi 6250, Bangladesh; n_islamm@yahoo.com

**Keywords:** antimicrobial drug, herbal medicine, medicinal plant, traditional medicine, *Vitex*

## Abstract

*Vitex* L. is the largest genus of the *Lamiaceae* family, and most of its species are used in the traditional medicinal systems of different countries. A systematic review was conducted, according to the PRISMA methodology, to determine the potential of *Vitex* plants as sources of antimicrobial agents, resulting in 2610 scientific publications from which 141 articles were selected. Data analysis confirmed that *Vitex* species are used in traditional medicine for symptoms of possible infectious diseases. Conducted studies showed that these medicinal plants exhibited *in vitro* antimicrobial activity against *Bacillus subtilis*, *Escherichia coli*, *Klebsiella pneumoniae*, *Pseudomonas aeruginosa,* and *Staphylococcus aureus*. *Vitex agnus-castus* L. and *Vitex negundo* L. have been the most studied species, not only against bacterial strains but also against fungi such as *Aspergillus niger* and *Candida albicans*, viruses such as HIV-1, and parasites such as *Plasmodium falciparum*. Natural products like agnucastoside, negundol, negundoside, and vitegnoside have been identified in *Vitex* extracts and their antimicrobial activity against a wide range of microbial strains has been determined. Negundoside showed significant antimicrobial activity against *Staphylococcus aureus* (MIC 12.5 µg/mL). Our results show that *Vitex* species are potential sources of new natural antimicrobial agents. However, further experimental studies need to be conducted.

## 1. Introduction

Bacterial resistance to clinically available antibiotics is a global phenomenon whose impact has increased significantly in recent years. Multidrug-resistant infections are a very common problem, greatly affecting mortality and morbidity in populations around the world and leading to greatly increased economic burdens. The Organization for Economic Cooperation and Development (OECD) estimates that the increase in multidrug-resistant bacterial infections will result in a total expense of approximately USD 20 to USD 35 trillion by 2050 [1,2]. Resistance mechanisms developed by bacteria to circumvent the effects of antibiotics are very diverse. Enzyme-based bacterial processes can directly inactivate antibiotics; efflux pumps can expel antibiotics from inside bacterial cells, reducing their concentration to subtoxic levels; mechanistic bacterial targets of antibiotics, such as ribosome subunits, DNA gyrase or RNA polymerase, can undergo conformational changes that prevent drugs from binding to them. Such mutations can be either spontaneous or adaptive, and some of them can undergo horizontal gene transfer, which can eventually lead to new naturally resistant bacteria. For example, *Staphylococcus aureus*, one of the most common etiological agents of infections in hospital and non-hospital contexts, shows a huge increase in resistance patterns to antibiotics of different classes, which means that this species can develop different resistance mechanisms to available drugs [2,3]. Medicinal plants have long been used in several traditional healing systems to treat many infectious symptoms and infectious diseases. Studies have shown that plant extracts (and/or natural products isolated from them) not only exert antimicrobial activity against various bacteria but can also modulate bacterial resistance mechanisms and increase the activity of concurrently administered antibiotics, or, in some cases, even reverse established resistance mechanisms. For example, several flavonoids, which constitute one of the most common classes of natural products, have demonstrated the ability to reverse bacterial multidrug resistance by inhibiting efflux pumps [4,5]. Research has shown that many plants’ secondary metabolites can exhibit antimicrobial activity, which can be exerted through a wide variety of mechanisms. Plants thus serve as direct antimicrobial agents and reservoirs of diverse bioactive compounds capable of inhibiting the growth and spread of harmful microorganisms. *Vitex* L., also known as the chaste tree genus, is the largest genus in the family *Lamiaceae* and comprises about 230 species distributed worldwide [6]. Most *Vitex* species are deciduous shrubs or small trees [7]. These species are scattered and mostly distributed in temperate regions of Asia and warm regions of Europe, being substantially distributed through Southeast Asia [8,9]. However, most species that belong to this genus are used in traditional medicine in southwest Asian countries like India, China, Nepal, Sri Lanka, Bangladesh, Malaysia, and other countries, namely Indonesia, Egypt, Iran, Morocco, Brazil, and Mexico. In India, *Vitex agnus-castus* L., *Vitex negundo* L., *Vitex peduncularis* W., *Vitex pinnata* L., and *Vitex trifolia* L. are frequently found throughout the country [10]. *Vitex* species are well recognized as sources of useful medicines in different geographic areas and have already been the subject of different research studies, mainly referring to *V. agnus-castus* and *V. negundo* [11].

Traditionally, *Vitex* plants have long been used for different types of treatment of menstrual disorders, fertility problems, menopausal symptoms, diarrhea, asthma, fever, cold, headache, migraine, gastrointestinal infections, and breast pain [12,13]. Recent studies have revealed that this genus has a wide range of biological properties, especially antimicrobial activities [14]. It has been utilized in various traditional medicinal systems around the world to address health concerns beyond its antimicrobial applications. Traditional practitioners usually prepare herbal medicines to treat and prevent diseases [15]. They use plant parts of *Vitex* species for the treatment of various infectious diseases such as bacterial, viral, and protozoal infections [16]. Several studies have examined the antimicrobial properties of *Vitex* L., and the results have shown that different parts of *Vitex* such as the leaf, bark, root, stem, flower, fruit, and seed exhibit antimicrobial activity against a wide range of microorganisms. Phytochemical analysis of the *Vitex* species has found several previously known compounds, mainly terpenoids, flavonoids, and alkaloids. This review has explored the potential antibacterial effects of *Vitex* extracts and their isolated natural products [17].

Herein, a concise and original review of the literature concerning the ethnomedicinal use of medicinal plants from the *Vitex* genus and their potential, as both antimicrobial herbal medicines as well as sources of new antimicrobial natural products, was made. This state-of-the-art paper will provide a comprehensive understanding of the potential of this genus as a source of antimicrobial agents.

## 2. Results

### 2.1. Selection of the Information

**Figure 1:**
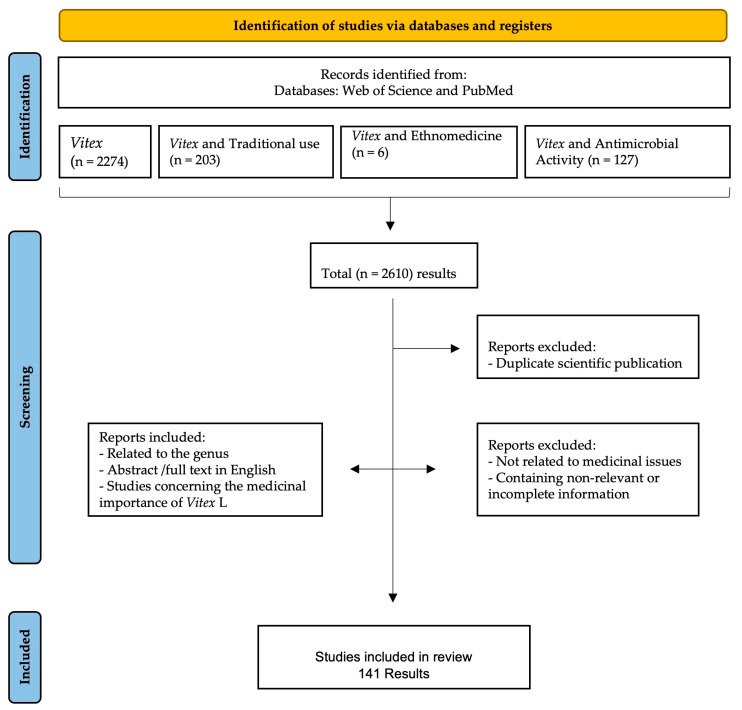
Data screening based on PRISMA methodology.

Details of data collection and selection are given in Figure 1. The initial title and abstract search yielded 2610 results. Of those, 2610 scientific publications were considered, and many articles were removed for the following reasons: repeated results, no relation to medicinal issues, and the inclusion of irrelevant or incomplete information. Finally, a total of 141 scientific publications were considered eligible to be included in this review as they were related to the use of *Vitex* species in traditional medicine, were abstracts or full texts written in English, and the studies conducted focused on the *Vitex* species and their antimicrobial activity against different microorganisms.

### 2.2. Traditional Uses

**Figure 2:**
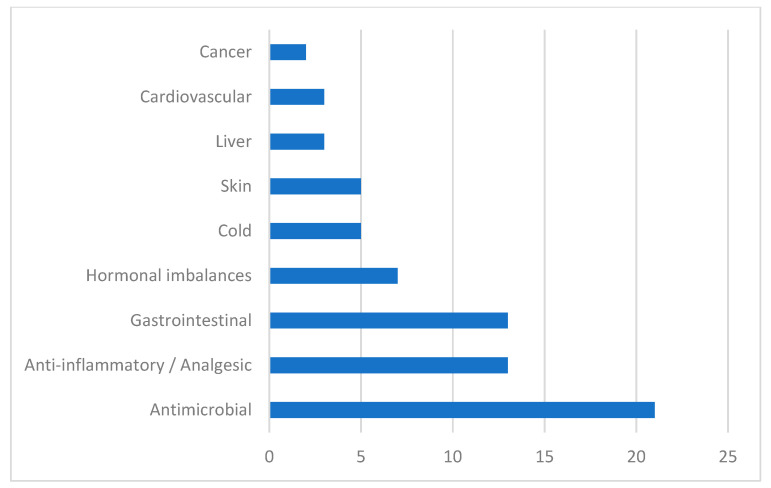
Symptoms of disease treated with *Vitex* plants.

Obtained results concerning the traditional use of the *Vitex* species are summarized in Table 1 and classified according to the symptoms they were used against (Figure 2**)**. From the recognized 230 species of *Vitex*, only 13 species have been reported as being used in traditional medicine, namely *Vitex agnus-castus* L., *Vitex doniana* L., *Vitex gardneriana* Schauer., *Vitex mollis* L. *Vitex negundo* L., *Vitex obovata* ssp. *wilmsii* (Gürke) Bredenkamp & Botha, *Vitex peduncularis* W., *Vitex peduncularis* L., *Vitex pinnata* L., *Vitex polygama* L., *Vitex pseudo-negundo* L., *Vitex rehmannii* sp., *Vitex rotundifolia* L., and *Vitex trifolia* L. These species are traditionally used for the treatment of menstrual disorders and hormonal imbalance, increasing breast milk production, and hypertension [18,19,20,21]. *Vitex* species are also used for infectious diseases treatment such as cavity infections, dysentery, diarrhea, asthma, cholera, and malaria [22,23,24,25,26]. The leaf of *Vitex* is the most frequently used plant part for medicinal purposes, but other parts like the bark, root, and flower are also referred to in the literature.

In Figure 2, we can see the major symptoms that are treated with *Vitex* species, grouped according to the physiological systems impaired. Results showed that these species are mostly used by traditional medical practitioners as antimicrobial agents. *Vitex* plants are also used in inflammatory diseases, as analgesics, as hormonal regulators, and in infectious and non-infectious gastrointestinal diseases.

### 2.3. In Vitro Antibacterial Activity Studies

Reviewed articles were screened for information regarding plant species and corresponding origin, plant parts and solvents used for extract preparation, antimicrobial activity essay performed, bacteria species used to evaluate antimicrobial activity, and substances used as control. Results were expressed as minimum inhibitory concentrations, minimum bactericidal concentrations, and inhibition zones exhibited according to the type of essay performed. The gathered information is summarized in Table 2. Our analysis showed that different essays were used to study antimicrobial activity against a wide variety of bacterial species and strains. *Bacillus subtilis*, *Escherichia coli*, *Klebsiella pneumoniae*, *Pseudomonas aeruginosa*, and *Staphylococcus aureus* were the most frequent subjects studied, mainly through disk diffusion and broth dilution methodology. Among the controls used in antimicrobial activity essays were known antibiotics like amoxicillin, chloramphenicol, ciprofloxacin, and gentamicin, and results showed that *Vitex* species plant extracts often exhibited significant activity against tested strains.

**Figure 3:**
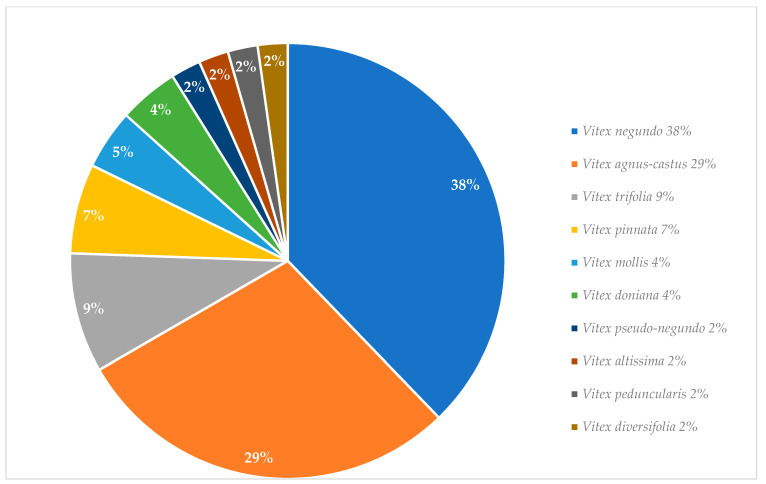
*Vitex* species studied for their *in vitro* antibacterial activity.

**Figure 4:**
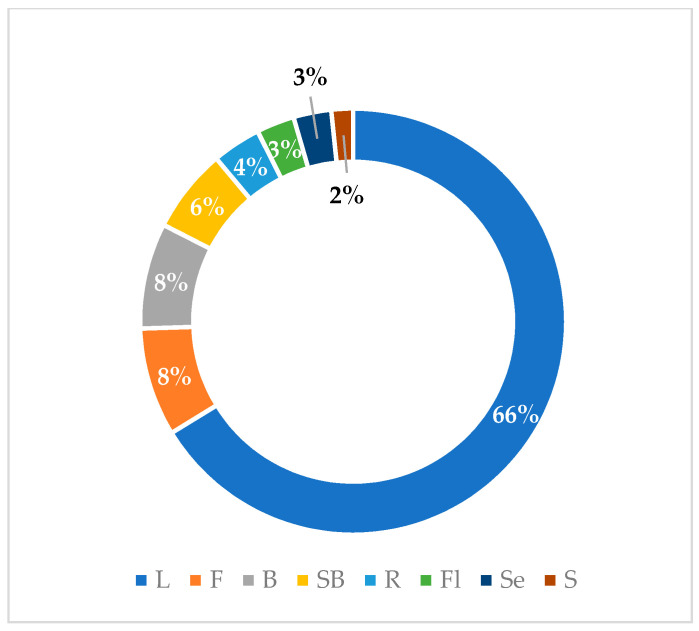
Plant parts used in antibacterial studies.

**Figure 5:**
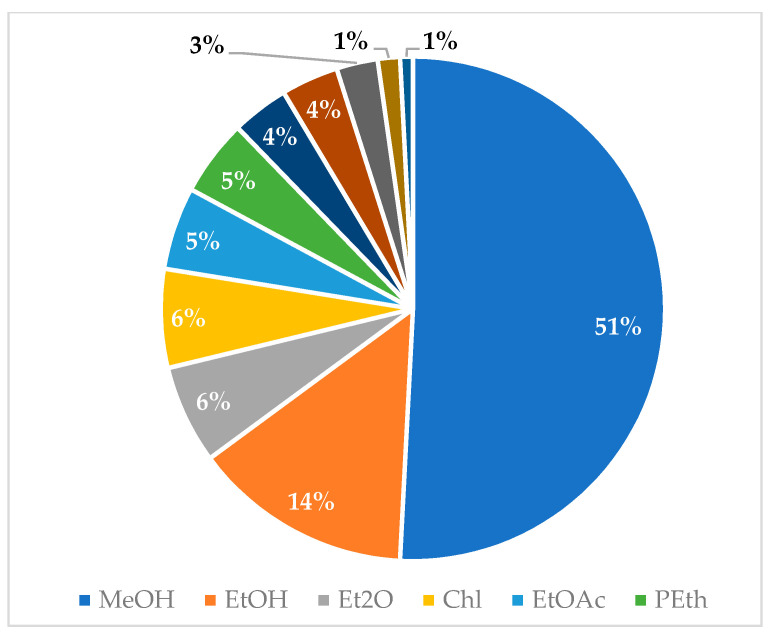
Solvents used for plant extraction for antibacterial activity essays.

**Figure 6:**
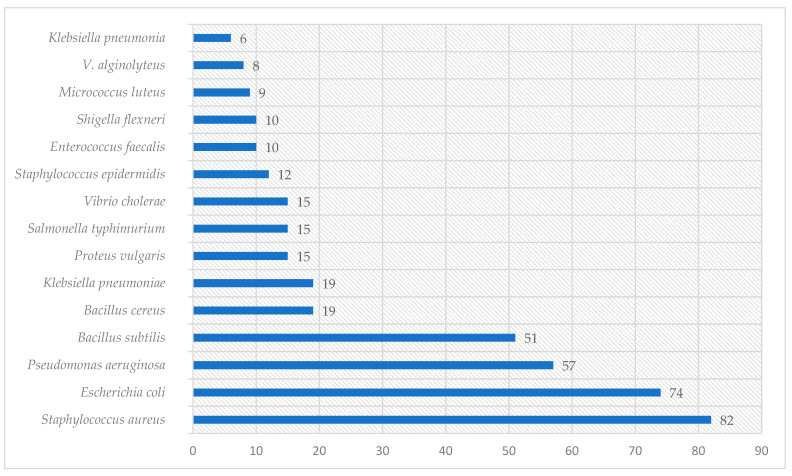
The microorganisms were extensively studied for their interactions with *Vitex* species.

Graphical interpretations of these results can be seen in Figure 3, Figure 4, Figure 5 and Figure 6. Figure 3 shows that *V. negundo* is the most studied *Vitex* species (38%), followed by *V. agnus-castus* (29%). Other species do not have the same expression in terms of scientific research focus. In Figure 4 and Figure 5, we can see that most of the studies focused on leaf plant parts and methanolic and ethanolic extracts of plant material. An analysis of Figure 6 shows the main bacterial strains that *Vitex* species have been tested on. These strains are widely known to be responsible for infections in humans, which makes the antibacterial activity exhibited by *Vitex* species an important focus of research for the development of new drugs.

Collected data show that all *Vitex* species used in traditional medicine to treat symptoms of infectious diseases exhibit *in vitro* antimicrobial activity against several bacterial strains, which can justify their use in traditional medicine to treat symptoms of infectious diseases.

### 2.4. In Vitro Antifungal, Antiviral, and Antiprotozoal Activity

Results showed that *Vitex* species exhibit biological activity, such as through antifungal, antiprotozoal, and antiviral activities. This information is summarized in Table 3 and Table 4*. V. negundo* and *V. agnus-castus* were the most studied plant species against a wider variety of these types of microorganisms. Methanolic extracts of leaf and root were the most frequent types of extract and plant parts used. Microbial agents tested were mostly fungal, namely *C. albicans* and *A. niger*, but viruses like HIV-1 and parasites like *Plasmodium falciparum* were also tested. Nevertheless, the most noteworthy significant value was observed in terms of antifungal activity against *C. albicans*. For example, an antifungal activity evaluation of ethanolic, methanolic, and aqueous extracts of the *V. agnus-castus* leaf showed that all had the ability to inhibit *Candida* species growth. Minimum inhibitory concentrations of studied extracts ranged from 25 µg/mL to 12.5 µg/mL against *C. tropicalis*, *C. albicans*, and *C. ciferri* while minimum fungicidal concentrations ranged from 100 µg/mL to 25 µg/mL [118].

### 2.5. Characteristic Vitex Secondary Metabolites with Antimicrobial Activity

**Figure 7:**
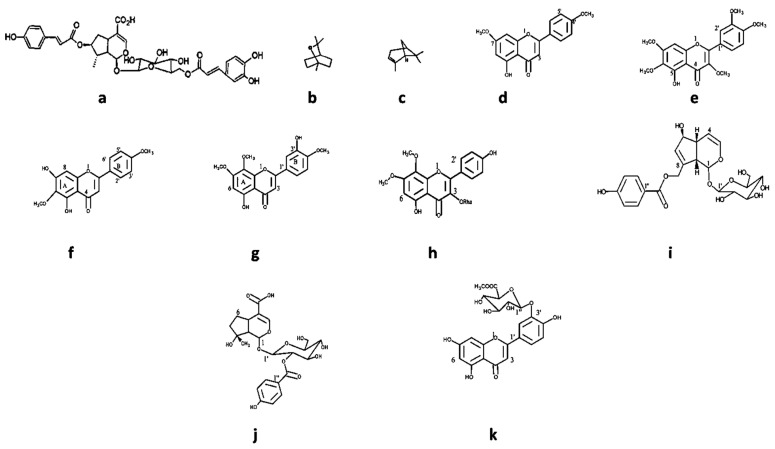
Some chemical structure of compounds: (**a**) agnucastoside, (**b**) 1,8-cineole, (**c**) α-pinene, (**d**) 5-hydroxy-7,4′dimethoxy flavone, (**e**) 5-hydroxy-3,6,7,3′,4′-pentamethoxy flavone, (**f**) 5,7 dihydroxy-6,4′ dimethoxy flavanone, (**g**) 5,3′ dihydroxy—7,8,4′-trimethoxy flavone, (**h**) 7,8 dimethyl herbacetin 3-rhamnoside, (**i**) agnuside, (**j**) negundoside, and (**k**) vitegnuside isolated from Vitex species.

Specific compounds have been isolated from *Vitex* species (Figure 7 and Table 5) and described for their antimicrobial activity. Most natural products were isolated from *V. negundo*. Different chemical classes such as phenolic compounds (like 5-hydroxy-7,4′ dimethoxy flavone) and terpenoids (like agnuside, negundoside, and vitegnoside) have been isolated from *Vitex* species [126,127]. Concerning the isolated natural products’ antimicrobial activity, negundoside is the most active one, showing significant antibacterial activity against *B. subtilis*, *E. coli*, *M. pyogenes*, *P. aeruginosa*, and *S. aureus*. Agnuside and vitegnoside, also isolated from *V. negundo*, showed a similar range of antibacterial activity against the same bacterial strains as negundoside. All these natural products were isolated from the methanolic leaf extract of the plant.

Isolated from *V. agnus-castus* fruit and leaf essential oils, α-pinene and 1,8-cineole exhibited significant *in vitro* antibacterial activity against *B. subtilis*, *E. coli*, *M. flavus*, *S. aureus*, and other strains. Both compounds were active in preventing *A. niger*-induced rotting in an *in vivo* apple fruit assay.

## 3. Materials and Methods

This review was performed following the criteria described in the Preferred Reporting Items for Systematic Reviews and Meta-Analyses (PRISMA) statement 2020 (http://www.prisma-statement.org/PRISMAStatement/FlowDiagram; accessed on 1 January 2022).

### 3.1. Search Strategy

The scientific data was collected from Web of Science and PubMed scientific publications that were published between 1 January 1980 and 23 May 2023, applying several keywords: *Vitex*, *Vitex* AND Traditional Use, *Vitex* AND Ethnomedicine, *Vitex* AND Biology, and *Vitex* AND Antimicrobial activity.

### 3.2. Data Inclusion and Exclusion Criteria

#### 3.2.1. Inclusion Criteria

- Related to the *Vitex* genus.
- Abstract or full text in English.
- Studies on *Vitex* species concerning antimicrobial activity.

#### 3.2.2. Exclusion Criteria

- Duplicate scientific publications.
- Not directly related to medicinal issues.
- Containing irrelevant or incomplete information.

## 4. General Discussion

Results of our study confirm that *Vitex* species have been used in traditional medicinal systems to approach several disease symptoms, the most frequent ones being related to infectious diseases, inflammatory states, gastrointestinal disorders, hormonal imbalances, cold symptoms, skin conditions, and liver and cardiovascular symptoms [18].

From more than 200 different *Vitex* species, 13 species were referred to in this review as the most used in traditional medicine; however, only 10 of them have been studied *in vitro* for their antimicrobial activity. *V. negundo* and *V. agnus-castus* are, by far, the two species that most frequently have been the focus of scientific research. The observed antimicrobial activity against a wide variety of microbial strains *in vitro* essays contributes to useful scientific validation for the main utilization of *Vitex* plants in traditional medicinal systems against infectious diseases. Even though specific mechanistic pathways that lead to bacterial death or growth inhibition are still unknown, the published literature indicates that these can be related to major constituents’ classes of natural products present in the corresponding tested extracts. [6].

In 2011, Kannathasan et al. conducted a study focusing on the antibacterial activity of several *Vitex* species. Leaves of *V. altissima*, *V. diversifolia*, *V. negundo*, *V. peduncularis,* and *V. trifolia* were used to prepare methanolic extracts, which were then evaluated for their antibacterial activity using the disc diffusion method. Results showed that *Vitex* extracts under analysis exhibited a wide range of activity against all tested microorganisms, like *S. aureus*, *E. coli*, *K. pneumoniae*, *P. aeruginosa*, and *P. mirabilis*. Mean zones of inhibition observed showed that the antibacterial activity of the extracts was selective for the microorganisms. Generally, *V. peduncularis* and *V. trifolia* extracts exhibited larger zones of inhibition than extracts of the other species against all microorganisms, being significantly active when compared to ciprofloxacin used as the control [137].

Research conducted by Berrani et al. on the phytochemical composition and biological activity of *V. negundo* showed that phenolic compounds were amongst the major constituents of the methanolic extracts analyzed. These extracts were prepared using leaf, root, stem, flower, and seed samples and tested separately against a panel of microbial agents commonly responsible for pathogenic infections in humans. Results showed that all plant extracts had selective antibacterial activity against all tested strains [74]. 

In a study conducted in 2010 by Nagarsekar et al., leaves of *V. negundo* were used to prepare different extracts. These extracts were studied for their antimicrobial activity against different microorganisms and characterized for their phytochemical composition. Different concentrations of ethanolic, petroleum ether, steam-distilled and supercritical fluid extracts were tested using the well diffusion method to evaluate their antimicrobial activity. Selective activity against *S. aureus* and *B. subtilis* and the increase in extract concentration were correlated with an increase in the exhibited antimicrobial activity [82].

Ababutain et al. studied ethanolic, methanolic, and aqueous leaf extracts of *V. agnus-castus* for their antifungal activity, using the agar well diffusion method, against *C. tropicalis*, *C. albicans*, and *C. ciferrii*. Tested extracts showed selective antifungal activity against *Candida* species. The aqueous extract was the most active one against all species, followed by the methanolic and ethanolic extracts. These results showed significant zones of inhibition when compared with the nystatin control [138].

Essential oils of the leaf and fruit of *V. agnus-castus* were characterized for their chemical composition in a work conducted by Stojkovic et al. in 2011. Obtained phytochemical profiles showed that 1,8-cineole and α-pinene were major constituents present in all tested essential oils. An evaluation of the antibacterial activity of the essential oils obtained from different plant parts was conducted using the microdilution method, testing against a panel of Gram-positive and Gram-negative bacteria. Selective activity was observed against *M. flavus*, *B. subtilis*, *S. typhimurium*, *S. aureus*, and *E. coli*, with all oils showing Minimum Inhibition Concentration (MIC) and Minimum Bactericidal Concentration (MBC) levels higher than the ones observed for the streptomycin control. The isolated compounds 1,8-cineole and α-pinene were also tested for antimicrobial activity. Both compounds exhibited significantly lower MICs and MBCs, not only compared to whole essential oils but also when compared to the streptomycin control, against all tested strains. The same behaviors were observed for all essential oils and isolated compounds against fungal pathogens like *A. alternata*, *A. flavus*, *A. niger*, *A. ochraceus*, *F. tricinctum*, *P. ochrochloron*, *P. funiculosum*, and *T. viride.* In this work, antifungal activity was also evaluated through an *in vivo* model of *A. niger-*induced rotting in apple fruits. Results showed that increasing concentrations of 1,8-cineole effectively reduced infectious disease incidence after 3 days of treatment [78].

It is well known that plant extracts are characterized by different natural products belonging to a wide variety of chemical classes of secondary metabolites. Phenolic compounds such as flavonoids constitute one of the most common chemical classes of secondary metabolites isolated from *Vitex* extracts and have previously been shown to exert antimicrobial activity through different mechanisms of action. For instance, apigenin, a very common flavonoid present in several plant species, can inhibit nucleic acid synthesis by binding to bacterial DNA gyrase. It can also induce bacterial cell lysis through membrane disruption (which leads to intracellular content leakage) and cell envelope synthesis inhibition, compromising structural integrity; apigenin also can inhibit biofilm formation and quorum sensing, two bacterial mechanisms with a high impact on infection prevalence and pathogenicity. Quercetin, also a very common phenolic compound, exhibits some similar behaviors. It can inhibit nucleic acid synthesis, which compromises bacterial metabolic viability. It is an active membrane disruptor and inhibitor of cell envelope synthesis. Quercetin can prevent efflux pump activity, reverting or preventing antibiotic resistance mechanisms, and can also directly inhibit bacterial toxins and enzymes [101].

Terpenoids and terpenoid derivatives also constitute a very common class of secondary metabolites, with known antimicrobial activity. Mechanistic pathways that lead to bacterial death or growth inhibition are yet to be determined, but research has shown that terpenoid compounds can inhibit oxygen uptake and oxidative phosphorylation, two metabolic processes crucial for bacterial survival. Monoterpenes carvacrol, thymol, menthol, and geraniol have exhibited antimicrobial activity against Gram-positive and Gram-negative bacteria. Geraniol can increase *Enterococcus aerogenes* susceptibility to antibiotics by inhibiting bacterial efflux pumps. Menthol and thymol are both active against *E. coli* and *S. aureus*. Carvacrol has been reported to inhibit the biofilm development of *S. aureus* and *S. typhimurium*. Oleanic acid, a triterpenoid, has shown antimicrobial activity against *M. tuberculosis* and a synergistic effect when administered with rifampicin, isoniazide, and ethambutol, significantly decreasing the MICs of these antibiotics [118]. 

The fact that most secondary metabolites isolated from *Vitex* plants are phenolic compounds or terpenoids falls in line with published literature focusing on the biological activity of these types of compounds and can be correlated with the exhibited antimicrobial activity. Most of the studies assessed in this review focused on methanolic and ethanolic extracts of the *Vitex* species’ leaf plant part. This may indicate that natural compounds present in this plant part are the main responsible ones for the antimicrobial activity exhibited and that these compounds must have high polarity rates since methanol and ethanol are polar solvents with high affinity for polar compounds. Regarding specific chemical compound isolation, studies conducted on *Vitex* species have identified diterpenoids and flavonoids. Since *Vitex* species have phytochemical profiles with high amounts of diterpenoids, these may be responsible for the exerted antimicrobial activity. However, further research should be conducted to better understand and characterize this activity. 

The drug development of new antimicrobial agents is a major challenge for the pharmaceutical industry. There are limited mechanistic pathways that can be followed for antibacterial, antifungal, and antiviral activities, and some microorganisms can have intrinsic specific resistances that render them immune to some of these pathways. Moreover, the development of new drugs “from scratch” is a highly expensive and time-consuming process that often does not move from theory to practice due to limitations like synthesis yields, formulation compatibilities, bioavailabilities, and other technological aspects. Using natural products from plants with antimicrobial activities can circumvent some of these limitations since pharmaceutical engineering processes can use core molecular skeletons of these secondary metabolites to develop new therapeutic options with clinical significance. For example, amikacin, a semisynthetic aminoglycoside broad-spectrum antibiotic active against *Pseudomonas aeruginosa* and most Gram-negative aerobes, is derived from kanamycin A, which is a natural product isolated from *Streptomyces kanamyceticus* [137,138,139].

Numerous studies have explored the efficacy of different plant parts of *Aloe vera*, namely leaf extracts, against a range of bacterial and fungal species. Results showed high antibacterial activity against *E. coli*, with inhibition zones indicating significant activity against drug-resistant strains [140]. On the other hand, *Artemisia* has shown antibacterial activity against drug-resistant bacterial strains. For example, *Artemisia absinthium* L. leaf extracts exhibit antibacterial activity against *S. aureus* [141]. Given the results of our review, we believe that the limited nature of the direct evidence on the antimicrobial activities of *Vitex* species, when compared to other genera, is due to the fact that *Vitex* species have been less extensively studied for their antimicrobial properties, and more research is needed to understand their full potential.

## 5. Conclusions

This review summarizes the main traditional uses of plants belonging to the *Vitex* genus as anti-infective medicines in different traditional medicinal systems and the *in vitro* antimicrobial activity demonstrated by extracts made from these medicinal plants against a wide variety of bacterial, fungal, and protozoal strains. *In vitro* studies demonstrate that *Vitex* extracts very often exhibit antimicrobial activity against different bacterial, fungal, and protozoal species. Some of the natural compounds present in these extracts, most likely the main ones, can be responsible for this biological activity. The results analyzed contribute to legitimizing the traditional use of *Vitex* species in traditional medicinal systems. A better understanding of *Vitex*-genus medicinal plants and their natural compounds can constitute a valuable natural source for discovering antimicrobial drugs and helping fight and prevent infectious diseases. 

## Figures and Tables

**Table 1:** Ethnomedicinal use of *Vitex* species.

Species	Part Used	Country	Signals or Symptoms or Pathology	Bib. References
*V. agnus-castus*	L	Iran	increasing breast milk	[18]
	F	Turkey	corpus luteum insufficiency, hyperprolactinemia, infertility, menstrual disorders, premenstrual dysphoric disorder, menopause disrupted lactation, cyclical gastralgia	[19,20]
	L	Brazil	oral disorders; diuretic, antiseptic, digestive, antifungal, anti-anxiety, aphrodisiac, anti-estrus, emmenagogus, antispasmodic, aperitif, and analgesic properties	[22]
	L	Brazil	menstrual disorder	[27]
*V. doniana*	Sb	Nigeria	decoction, gastroenteritis, diarrhea, dysentery	[25]
*V. gardneriana*	L	Brazil	analgesic pain, anti-inflammatory properties	[28]
*V. mollis*	L	Mexico	dysentery, analgesic, anti-inflammatory properties, scorpion stings, diarrhea, stomachache	[29]
*V. negundo*	S, F	India	increasing lactation	[30,31]
	L	India	post-partum bath	[30,31]
	R, B, Fl	India	diarrhea, dysentery, flatulence, indigestion, cholera	[24,32]
	L	Maldives	fever	[33]
	L	Bangladesh, India, Malaysia	headache	[26,34]
	L	China, India, Nepal	cough, sore throat	[35,36,37]
	R, L	India	rheumatism	[38]
	L	India	hives, cellulitis, carbuncle	[39]
	L	India	fever, hearing problems	[24,40]
	Fl	Philippines	cancer	[41]
	L	Bangladesh	chronic disease, infectious diseases	[42]
	L	India	paralysis	[43]
	L	China	skin disease	[44]
	L	China	coughs, phlegm, asthma	[45]
	L	Pakistan	antiallergic properties	[46]
	R, Wp	Bangladesh	malaria, fever	[26]
	L	China and India	stomachic, antiseptic, depurative, and rejuvenating properties; eye problems; gonorrhea	[47]
	L	Bangladesh	diarrhea, dysentery	[48]
	L, B	Nepal	jaundice, wounds, body ache, toothache, asthma, eye problems	[49]
*V. obovata*	L	South Africa	body pain	[50]
*V. peduncularis*	Wp	India	wounds, dysentery, stomach diseases, fever, hypertension	[51]
	B, L	Bangladesh	joint ache, diabetes	[26]
	R	India	eye problems, skin problems, chest pain	[52]
	L	India	malaria, fever	[53]
*V. pinnata*	Wp	Malaysia	hypertension, gastrointestinal disorders	[21]
	L	Brunei	hypertension, fever	[16]
	L, B	Malaysia	fever, gastric ulcer	[54]
	Wp	Brunei	jaundice	[55]
	L	Brunei	sanitizing	[23]
	Wp	Malaysia	dysentery, inflammatory	[56]
	Wp	Indonesia	cancer, gastrointestinal disorder, fever, wound, skin tumor	[57]
*V. polygama*	L, F	Brazil	emmenagogue and diuretic properties.	[58]
*V. pseudo-negundo*	L	Iran	hyperprolactinemia, hormonal imbalance syndromes, breast diseases, infertility	[59]
*V. rehmannii*	L	South Africa	stomach disease	[50]
*V. rotundifolia*	F	China	cold, headache	[60]
*V. trifolia*	L	India	liver disorders, rheumatic pains	[61]
	L	India	ulcers	[62]
	L	Philippines	cough	[63]
	F	China	migraines, eye problems	[45]
	Fl	Bangladesh	fever, vomiting	[64]
	L	Fiji	coughs, gonorrhea, stomach pain	[64]
	L	Tongo	infections	[65]
	St, L	Madagascar	stomach pain	[66]
	Fl	Thailand	asthma	[67]

B—Bark; F—Fruit; Fl—Flower; L—Leaf; R—Root; Sb—Steambark; St—Steam; Wp—Whole plant.

**Table 2:** *In vitro* antibacterial activity studies on *Vitex* species.

Species	Country	Plant Part Used	Extractive Solvent/Compound	Test Type	Strains	Positive Control	Results	R
*V.* *agnus-castus* *castus7*	Iran	F	H_2_O	DDM	*Bacillus cereus* PTCC 1015 *Escherichia coli* PTCC 1399	gentamicin and ciprofloxacin	IZ 5 mm	[68]
	Iran	F	H_2_O	BDM	*Escherichia coli* PTCC 1399	gentamicin and ciprofloxacin	MIC 25 µg/mL	[68]
	Iran	F	H_2_O	BDM	*Bacillus cereus* PTCC 1015	gentamicin and ciprofloxacin	na	[68]
	Iran	F	H_2_O	BDM	*Escherichia coli* PTCC 1399	gentamicin and ciprofloxacin	MIC 12 µg/mL	[68]
	Iran	F	H_2_O	BDM	*Bacillus cereus* PTCC 1015	gentamicin and ciprofloxacin	MIC 25 µg/mL	[68]
	Egypt	L	Et_2_O	ADM	*Agrobacterium tumefaciens* *	na	MIC 575 mg/L	[69]
	Egypt	L	Et_2_O	ADM	Erwinia carotovora var. carotovora *	na	MIC 425 mg/L	[69]
	Brazil	L	EtOAc	BMicDM	*Streptococcus mutans* ATCC 25175 *Lactobacillus casei* ATCC 11578	chlorhexidine dihydrochloride	MIC 15.6 µg/mL	[22]
	Brazil	L	EtOAc	BMicDM	*Streptococcus mitis* ATCC 49456	chlorhexidine dihydrochloride	MIC 31.25 µg/mL	[22]
	Brazil	L	EtOAc	BMicDM	*Streptococcus subrinus* ATCC 33478	chlorhexidine dihydrochloride	MIC 125 µg/mL	[22]
	Brazil	L	EtOAc	BMicDM	*Streptococcus salivarius* ATCC 25975	chlorhexidine dihydrochloride	MIC 200 µg/mL	[22]
	Bulgaria	F	Et_2_O	AWDM	*Staphylococcus aureus* ATCC 6538	na	IZ 11.25 ± 0.05 mm	[70]
	Bulgaria	F	Et_2_O	AWDM	*Bacillus subtilis* ATCC 6633	na	IZ 12.03 ± 0.02 mm	[70]
	Bulgaria	F	Et_2_O	AWDM	*Kocuria rhizophila* ATCC 9341	na	IZ 9.37 ± 0.04 mm	[70]
	Bulgaria	F	Et_2_O	AWDM	*Escherichia coli* ATCC 8739	na	IZ 8.00 ± 0.0 mm	[70]
	Bulgaria	F	Et_2_O	AWDM	*Pseudomonas aeruginosa* ATCC 9027	na	IZ 8.03 ± 0.02 mm	[70]
	Bulgaria	F	Et_2_O	AWDM	*Salmonella abony* NCTC 6017	na	IZ 11.15 ± 0.05 mm	[70]
	Bulgaria	F	Et_2_O	AWDM	*Saccharomyces cerevisiae* ATCC 2601	na	IZ 11.86 ± 0.03 mm	[70]
	Egypt	L	Et_2_O	DDM	*Staphylococcus aureus* ATCC 6358	chloramphenicol	IZ 30 mm	[71]
	Egypt	L	Et_2_O	DDM	*Bacillus subtilis* ATCC 6633	chloramphenicol	IZ 10 mm	[71]
	Egypt	L	Et_2_O	DDM	*Escherichia coli* ATCC 25923	chloramphenicol	IZ 20 mm	[71]
	Egypt	L	Et_2_O	DDM	*Pseudomonas aeruginosa* ATCC 27853	chloramphenicol	IZ 20 mm	[71]
	Turkey	Fl	MeOH	DDM	*Staphylococcus aureus* 17	ampicillin 10 µg and oxacillin 5 ug	IZ 18 mm	[72]
	Turkey	Fl	MeOH	DDM	*Staphylococcus aureus* 18	ampicillin 10 µg and oxacillin 5 ug	IZ 12 mm	[72]
	Turkey	Fl	EtOH	DDM	*Coagulate negative Staphylococci* 33	ampicillin 10 µg and oxacillin 5 ug	IZ 8 mm	[72]
	Turkey	Fl	MeOH	DDM	*Coagulate negative Staphylococci* 33	ampicillin 10 µg and oxacillin 5 ug	IZ 10 mm	[72]
	Turkey	Fl	EtOH	DDM	*Coagulate negative Staphylococci* 36	ampicillin 10 µg and oxacillin 5 ug	IZ 9 mm	[72]
	Turkey	L	EtOH	DDM	Staphylococcus aureus *	gentamycin	IZ 7.5 mm	[73]
	Turkey	F	EtOH	DDM	Staphylococcus aureus *	gentamycin	IZ 10 mm	[73]
	Turkey	L	EtOH	DDM	Pseudomonas aeruginosa *	gentamycin	IZ 9 mm	[73]
	Morocco	L	MeOH	BMicDM	*Bacillus subtilis* CIP 5262	chloramphenicol	MIC 15.62 µg/mL MBC 15.62 µg/mL	[74]
	Morocco	R	MeOH	BMicDM	*Bacillus subtilis* CIP 5262	chloramphenicol	MIC 31.25 µg/mL MBC 31.25 µg/mL	[74]
	Morocco	S	MeOH	BMicDM	*Bacillus subtilis* CIP 5262	chloramphenicol	MIC 15.62 µg/mL MBC 15.62 µg/mL	[74]
	Morocco	Fl	MeOH	BMicDM	*Bacillus subtilis* CIP 5262	chloramphenicol	MIC 15.62 µg/mL MBC 31.25 µg/mL	[74]
	Morocco	Se	MeOH	BMicDM	*Bacillus subtilis* CIP 5262	chloramphenicol	MIC 15.62 µg/mL MBC 15.62 µg/mL	[74]
	Morocco	L	MeOH	BMicDM	*Escherichia coli* CIP 53126	chloramphenicol	MIC 15.62 µg/mL MBC 15.62 µg/mL	[74]
	Morocco	R	MeOH	BMicDM	*Escherichia coli* CIP 53126	chloramphenicol	MIC 15.62 µg/mL MBC 15.62 µg/mL	[74]
	Morocco	S	MeOH	BMicDM	*Escherichia coli* CIP 53126	chloramphenicol	MIC 15.62 µg/mL MBC 31.25 µg/mL	[74]
	Morocco	Fl	MeOH	BMicDM	*Escherichia coli* CIP 53126	chloramphenicol	MIC 31.25 µg/mL MBC 31.25 µg/mL	[74]
	Morocco	S	MeOH	BMicDM	*Escherichia coli* CIP 53126	chloramphenicol	MIC 15.62 µg/mL MBC 15.62 µg/mL	[74]
	Morocco	L	MeOH	BMicDM	*Pseudomonas aeruginosa* CIP 82118	chloramphenicol	MIC 15.62 µg/mL MBC 15.62 µg/mL	[74]
	Morocco	R	MeOH	BMicDM	*Pseudomonas aeruginosa* CIP 82118	chloramphenicol	MIC 15.62 µg/mL MBC 15.62 µg/mL	[74]
	Morocco	S	MeOH	BMicDM	*Pseudomonas aeruginosa* CIP 82118	chloramphenicol	MIC 15.62 µg/mL MBC 15.62 µg/mL	[74]
	Morocco	Fl	MeOH	BMicDM	*Pseudomonas aeruginosa* CIP 82118	chloramphenicol	MIC 7.81 µg/mL MBC 7.81 µg/mL	[74]
	Morocco	Se	MeOH	BMicDM	*Pseudomonas aeruginosa* CIP 82118	chloramphenicol	MIC 15.62 µg/mL MBC 15.62 µg/mL	[74]
	Morocco	L	MeOH	BMicDM	*Salmonella enterica* CIP 8039	chloramphenicol	MIC 7.81 µg/mL MBC 15.62 µg/mL	[74]
	Morocco	R	MeOH	BMicDM	*Salmonella enterica* CIP 8039	chloramphenicol	MIC 31.25 µg/mL MBC 31.25 µg/mL	[74]
	Morocco	S	MeOH	BMicDM	*Salmonella enterica* CIP 8039	chloramphenicol	MIC 7.81 µg/mL MBC 15.62 µg/mL	[74]
	Morocco	Fl	MeOH	BMicDM	*Salmonella enterica* CIP 8039	chloramphenicol	MIC 7.81 µg/mL MBC 15.62 µg/mL	[74]
	Morocco	Se	MeOH	BMicDM	*Salmonella enterica* CIP 8039	chloramphenicol	MIC 7.81 µg/mL MBC 15.62 µg/mL	[74]
	Morocco	L	MeOH	BMicDM	*Staphylococcus aureus* CIP 483	chloramphenicol	MIC 15.62 µg/mL MBC 15.62 µg/mL	[74]
	Morocco	R	MeOH	BMicDM	*Staphylococcus aureus* CIP 483	chloramphenicol	MIC 31.25 µg/mL MBC 31.25 µg/mL	[74]
	Morocco	S	MeOH	BMicDM	*Staphylococcus aureus* CIP 483	chloramphenicol	MIC 15.62 µg/mL MBC 15.62 µg/mL	[74]
	Morocco	Fl	MeOH	BMicDM	*Staphylococcus aureus* CIP 483	chloramphenicol	MIC 7.81 µg/mL MBC 15.62 µg/mL	[74]
	Morocco	Se	MeOH	BMicDM	*Staphylococcus aureus* CIP 483	chloramphenicol	MIC 15.62 µg/mL MBC 15.62 µg/mL	[74]
	Turkey	F	MeOH	DDM	*Bacillus subtilis* ATCC 6051	ampicillin	IZ 25 mm	[75]
	Turkey	F	MeOH	DDM	*Bacillus subtilis* ATCC 6051	ofloxacin	IZ 30 mm	[75]
	Turkey	F	MeOH	DDM	*Escherichia coli* ATCC 11775	ampicillin	IZ 28 mm	[75]
	Turkey	F	MeOH	DDM	*Escherichia coli* ATCC 11775	ofloxacin	IZ 28 mm	[75]
	Turkey	F	MeOH	DDM	*Enterococcus faecalis* ATCC 292	ampicillin	IZ 26 mm	[75]
	Turkey	F	MeOH	DDM	*Enterococcus faecalis* ATCC 292	ofloxacin	IZ 20 mm	[75]
	Turkey	F	MeOH	DDM	*Pseudomonas aeruginosa* ATCC 1014	ampicillin	IZ 23 mm	[75]
	Turkey	F	MeOH	DDM	*Pseudomonas aeruginosa* ATCC 1014	ofloxacin	IZ 23 mm	[75]
	Turkey	F	MeOH	DDM	*Staphylococcus aureus* ATCC 12600	ampicillin	IZ 20 mm	[75]
	Turkey	F	MeOH	DDM	*Staphylococcus aureus* ATCC 12600	ofloxacin	IZ 26 mm	[75]
	Turkey	F	MeOH	DDM	*Salmonella typhimurium* ATCC 2524	ampicillin	IZ 22 mm	[75]
	Turkey	F	MeOH	DDM	*Salmonella typhimurium* ATCC 2524	ofloxacin	IZ 24 mm	[75]
	Turkey	Se	MeOH	DDM	Escherichia coli *	erythromycin	IZ 16 mm	[76]
	Turkey	Se	MeOH	DDM	Staphylococcus aureus *	erythromycin	IZ 16 mm	[76]
	Lebanon	Fl	EtOAc	BMicDM	*Escherichia coli* ATCC 25922	oxacillin and gentamicin	MIC 512 µg/mL	[77]
	Lebanon	Fl	EtOAc	BMicDM	*Staphylococcus aureus* ATCC 29213	oxacillin and gentamicin	MIC 512 µg/mL	[77]
	Lebanon	Fl	EtOAc	BMicDM	*Candida albicans* ATCC 10231	oxacillin and gentamicin	MIC 512 µg/mL	[77]
	Lebanon	Fl	EtOAc	BMicDM	*Trichophyton rubrum* SNB-TR	oxacillin and gentamicin	MIC 512 µg/mL	[77]
	Serbia	L	EtOH	BMicDM	*Salmonella typhimurium* ATCC 13311	streptomycin	MIC 44.5 ± 0.9 µg/mL MBC 89.0 ± 1.5 µg/mL	[78]
	Serbia	L	EtOH	BMicDM	*Escherichia coli* ATCC 35210	streptomycin	MIC 219.0 ± 3.0 µg/mL MBC 445.0 ± 2.9µg/mL	[78]
	Serbia	L	EtOH	BMicDM	*Staphylococcus aureus* ATCC 6538	streptomycin	MIC 219.0 ± 1.7 µg/mL MBC 445.0 ± 5.8 µg/mL	[78]
	Serbia	L	EtOH	BMicDM	*Micrococcus flavus* ATCC 9341	streptomycin	MIC 445.0 ± 5.5 µg/mL MBC 890.0 ± 23.1 µg/mL	[78]
	Serbia	L	EtOH	BMicDM	*Bacillus subtilis* ATCC 10907	streptomycin	MIC 890.0 ± 11.0 µg/mL MBC 890.0 ± 5.8 µg/mL	[78]
	Iran	F	na	AWDM	Staphylococcus aureus *	gentamicin	MIC 62.5 ± 4.0 µg/mL MBC 125.0 ± 8.0 µg/mL	[79]
	Turkey	L	Et_2_O	BDM	*Staphylococcus aureus* ATCC 29213	ceftriaxone	MIC 22.8 µg/mL MBC 55.0 µg/mL	[80]
	Turkey	L	Et_2_O	BDM	*Staphylococcus aureus* ATCC BAA-977	ceftriaxone	MIC 13.7 µg/mL MBC 45.8 µg/mL	[80]
	Turkey	L	Et_2_O	BDM	*Enterococcus eliflavus* ATCC 700327	ceftriaxone	MIC 13.7 µg/mL MBC 27.5 µg/mL	[80]
	Turkey	L	Et_2_O	BDM	*Enterococcus faecalis* ATCC 29212	ceftriaxone	MIC 13.7 µg/mL MBC 27.5 µg/mL	[80]
	Turkey	L	Et_2_O	BDM	*Escherichia coli* ATCC 25922	ceftriaxone	MIC 27.5 µg/mL MBC 27.5 µg/mL	[80]
	Turkey	L	Et_2_O	BDM	*Pseudomonas aeruginosa* ATCC 27853	ceftriaxone	MIC 27.5 µg/mL MBC 55.0 µg/mL	[80]
	Turkey	L	Et_2_O	BDM	*Klebsiella pneumoniae* ATCC 700603	ceftriaxone	MIC 36.6 µg/mL MBC 55.0 µg/mL	[80]
	Turkey	L	Et_2_O	BDM	*Enterobacter hormaechei* ATCC 700323	ceftriaxone	MIC 27.5 µg/mL MBC 27.5 µg/mL	[80]
	Ukraine	L	EtOH	AWDM	Escherichia coli *	azithromycin	IZ 2.8 ± 0.17 mm	[81]
*V. altissima*	India	L	MeOH	BMacDM	*Bacillus cereus* NCIM 2155	ciprofloxacin	MIC 500 µg/mL MBC 1000 µg/mL IZ 13.500 ± 0.866 mm	[82]
	India	L	MeOH	BMacDM	*Bacillus pumilus* NCIM 2327	ciprofloxacin	MIC 500 µg/mL MBC 1000 µg/mL IZ 12.330 ± 0.258 mm	[82]
	India	L	MeOH	BMacDM	*Bacillus subtilis* NCIM 2063	ciprofloxacin	MIC 500 µg/mL MBC 1000 µg/mL IZ 13.160 ± 0.763 mm	[82]
	India	L	MeOH	BMacDM	*Micrococcus luteus* NCIM 2376	ciprofloxacin	MIC 125 µg/mL MBC 250 µg/mL IZ 14.800 ± 0.793 mm	[82]
	India	L	MeOH	BMacDM	*Staphylococcus aureus* NCIM 2901	ciprofloxacin	MIC 250 µg/mL MBC 500 µg/mL IZ 14.770 ± 0.437 mm	[82]
	India	L	MeOH	BMacDM	*Escherichia coli* NCIM 2256	ciprofloxacin	MIC 2000 µg/mL MBC 4000 µg/mL IZ 8.700 ± 0.435 mm	[82]
	India	L	MeOH	BMacDM	*Klebsiella pneumoniae* NCIM 2957	ciprofloxacin	MIC 2000 µg/mL MBC 4000 µg/mL IZ 8.270 ± 0.801 mm	[82]
	India	L	MeOH	BMacDM	*Pseudomonas aeruginosa* NCIM 5031	ciprofloxacin	MIC 2000 µg/mL MBC 4000 µg/mL IZ 8.130 ± 0.814 mm	[82]
	India	L	MeOH	BMacDM	*Proteus vulgaris* NCIM 2027	ciprofloxacin	MIC 2000 µg/mL MBC 4000 µg/mL IZ 9.360 ± 0.437 mm	[82]
	India	L	MeOH	BMacDM	*Salmonella typhimurium* NCIM 2501	ciprofloxacin	MIC 2000 µg/mL MBC 4000 µg/mL IZ 8.390 ± 0.437 mm	[82]
	India	L	MeOH	BMacDM	*Shigella flexneri* MTCC 1457	ciprofloxacin	MIC 1000 µg/mL MBC 2000 µg/mL IZ 11.180 ± 0.822 mm	[82]
	India	L	MeOH	BMacDM	*Shigella sonnei* MTCC 2597	ciprofloxacin	MIC 1000 µg/mL MBC 2000 µg/mL IZ 11.760 ± 0.473 mm	[82]
*V* *. diversifolia*	India	L	MeOH	BMacDM	*Bacillus cereus* NCIM 2155	ciprofloxacin	MIC 250 µg/mL MBC 500 µg/mL IZ 14.430 ± 0.473 mm	[82]
	India	L	MeOH	BMacDM	*Bacillus pumilus* NCIM 2327	ciprofloxacin	MIC 500 µg/mL MBC 1000 µg/mL IZ 13.590 ± 0.452 mm	[82]
	India	L	MeOH	BMacDM	*Bacillus subtilis* NCIM 2063	ciprofloxacin	MIC 5000 µg/mL MBC 1000 µg/mL IZ 13.740 ± 0.444 mm	[82]
	India	L	MeOH	BMacDM	*Micrococcus luteus* NCIM 2376	ciprofloxacin	MIC 125 µg/mL MBC 250 µg/mL IZ 16.180 ± 0.822 mm	[82]
	India	L	MeOH	BMacDM	*Staphylococcus aureus* NCIM 2901	ciprofloxacin	MIC 500 µg/mL MBC 250 µg/mL IZ 16.320 ± 0.435 mm	[82]
	India	L	MeOH	BMacDM	*Escherichia coli* NCIM 2256	ciprofloxacin	MIC 4000 µg/mL MBC 1000 µg/mL IZ 10.400 ± 0.525 mm	[82]
	India	L	MeOH	BMacDM	*Klebsiella pneumoniae* NCIM 2957	ciprofloxacin	MIC 4000 µg/mL MBC 1000 µg/mL IZ 10.150 ± 0.581 mm	[82]
	India	L	MeOH	BMacDM	*Pseudomonas aeruginosa* NCIM 5031	ciprofloxacin	MIC 4000 µg/mL MBC 2000 µg/mL IZ 8.810 ± 0.815 mm	[82]
	India	L	MeOH	BMacDM	*Proteus vulgaris* NCIM 2027	ciprofloxacin	MIC 4000 µg/mL MBC 2000 µg/mL IZ 10.180 ± 0.822 mm	[82]
	India	L	MeOH	BMacDM	*Salmonella typhimurium* NCIM 2501	ciprofloxacin	MIC 4000 µg/mL MBC 2000 µg/mL IZ 9.760 ± 0.473 mm	[82]
	India	L	MeOH	BMacDM	*Shigella flexneri* MTCC 1457	ciprofloxacin	MIC 2000 µg/mL MBC 1000 µg/mL IZ 11.250 ± 0.452 mm	[82]
	India	L	MeOH	BMacDM	*Shigella sonnei* MTCC 2597	ciprofloxacin	MIC 2000 µg/mL MBC 1000 µg/mL IZ 12.120 ± 0.785 mm	[82]
*V. doniana*	Nigeria	Sb	MeOH	BDM	*Escherichia coli ATCC 25922*	tetracycline	MIC > 500 µg/mL	[83]
	Nigeria	Sb	MeOH	ADM	Salmonella typhi *	na	MIC 0.31–2.5 µg/mL	[25]
	Nigeria	Sb	MeOH	ADM	Shigella dysentarae *	na	MIC 0.02–0.08 µg/mL	[25]
	Nigeria	Sb	MeOH	ADM	Escherichia coli *	na	MIC 0.04–0.38 µg/mL	[25]
	Nigeria	L	Et_2_O	DDM	*Bacillus subtilis* ATTC 33923	gentamicin	IZ 40 mm	[84]
	Nigeria	L	Et_2_O	DDM	*Staphylococcus aureus* ATTC 6538	gentamicin	IZ 36 mm	[84]
	Nigeria	L	Et_2_O	DDM	*Pseudomonas aeruginosa* ATTC 27856	gentamicin	IZ 36 mm	[84]
	Nigeria	L	Et_2_O	DDM	*Bacillus cereus* ATTC 14579	gentamicin	IZ 31 mm	[84]
	Nigeria	L	Et_2_O	DDM	*Proteus mirabilis* ATTC 21784	gentamicin	IZ 31 mm	[84]
*V. gardneriana*	Brazil	L	Et_2_O		*Staphylococcus aureus* ATCC 25923	na	MIC 0.31 µg/mL	[85]
*V. mollis*	Mexico	F	H_2_O	BMicDM	*Staphylococcus aureus* ATCC 29213	gentamicin	IZ 8.8 ± 0.26 mm	[86]
	Mexico	F	H_2_O	BMicDM	*Escherichia coli* A011	gentamicin	IZ 9.8 ± 0.35 mm	[86]
	Mexico	F	H_2_O	BMicDM	*Escherichia coli* A055	gentamicin	IZ 9.5 ± 0.70 mm	[86]
	Mexico	F	H_2_O	BMicDM	Shigella dysenteriae *	gentamicin	IZ 7.5 ± 0.70 mm	[86]
	Mexico	F	H_2_O	BMicDM	*Pseudomonas aeruginosa* ATCC 27853	gentamicin	IZ 8.8 ± 0.35 mm	[86]
	Mexico	F	H_2_O	BMicDM	*Escherichia coli* ATCC 25922	gentamicin	IZ 10 ± 1.41 mm	[86]
	Mexico	F	H_2_O	BMicDM	*Escherichia coli* ATCC 25922	gentamicin	MIC 7.5 µg/mL MBC 7.5 µg/mL	[86]
	Mexico	L	MeOH	DDM	*Staphylococcus aureus* ATCC *25923*	methicillin	IZ 14.50 ± 0.30 mm	[87]
	Mexico	L	MeOH	DDM	*Staphylococcus aureus* ATCC 29213	methicillin	IZ 14.63 ± 0.51 mm	[87]
	Mexico	L	MeOH	DDM	*Staphylococcus aureus* ATCC 43300	methicillin	IZ 12.37 ± 0.40 mm	[87]
	Mexico	L	MeOH	DDM	*Staphylococcus aureus* MRSA1	methicillin	IZ 12.43 ± 0.45 mm	[87]
	Mexico	L	MeOH	DDM	*Staphylococcus aureus* MRSA2	methicillin	IZ 18.53 ± 0.40 mm	[87]
	Mexico	L	MeOH	DDM	*Staphylococcus aureus* SOSA1	oxacillin	IZ 14.34 ± 0.30 mm	[87]
	Mexico	L	MeOH	DDM	*Staphylococcus aureus* SOSA2	oxacillin	IZ 18.60 ± 0.23 mm	[87]
	Mexico	L	MeOH	DDM	*Staphylococcus epidermidis* CoNS1	oxacillin	IZ 18.60 ± 0.23 mm	[87]
	Mexico	L	MeOH	DDM	*Staphylococcus epidermidis* CoNS2	oxacillin	IZ 14.25 ± 0.30 mm	[87]
	Mexico	L	MeOH	DDM	*Staphylococcus epidermidis* CoNS3	oxacillin	IZ 18.53 ± 0.50 mm	[87]
	Mexico	F	Chl	DDM	*Escherichia coli* ATCC 25922	ampicillin	MIC 2.0 μg/mL MBC 2.0 μg/mL	[88]
*V. negundo*	India	L	Chl	AWDM	*Staphylococcus aureus* *	gentamycin	IZ 21 mm	[89]
	India	L	Chl	AWDM	Bacillus subtilis *	tetracycline	IZ 18 mm	[89]
	India	L	Ace	AWDM	Staphylococcus aureus *	gentamycin	IZ 21 mm	[89]
	India	L	Ace	AWDM	Bacillus subtilis *	tetracycline	IZ 24 mm	[89]
	India	L	Ace	AWDM	Pseudomonas aeruginosa *	gentamycin	IZ 19 mm	[89]
	India	L	MeOH	AWDM	Staphylococcus aureus *	gentamycin	IZ 24 mm	[89]
	India	L	MeOH	AWDM	Pseudomonas aeruginosa *	gentamycin	MIC 0.078 µg/mL	[89]
	India	L	MeOH	AWDM	Pseudomonas aeruginosa *	gentamycin	IZ 18 mm	[89]
	India	L	MeOH	AWDM	Staphylococcus aureus *	gentamycin	IZ 21 mm	[89]
	India	L	MeOH	AWDM	Klebsiella pneumoniae *	ciprofloxacin	IZ 16 mm	[89]
	India	L	EtOH	AWDM	Staphylococcus aureus *	ciprofloxacin	IZ 11.4 ± 0.23 mm MIC 12.5 µg/mL	[90]
	India	L	EtOH	AWDM	Streptococcus epidermidis *	ciprofloxacin	IZ 12.4 ± 0.14 mm MIC 6.25 µg/mL	[90]
	India	L	EtOH	AWDM	Bacillus cereus *	ciprofloxacin	IZ 14.2 ± 0.14 mm MIC 25 µg/mL	[90]
	India	L	EtOH	AWDM	Corynebacterium xerosis *	ciprofloxacin	IZ 13.53 ± 0.14 mm MIC 50 µg/mL	[90]
	India	L	EtOH	AWDM	Escherichia coli *	gentamycin	IZ 14.0 ± 0.14 mm MIC 25 µg/mL	[90]
	India	L	EtOH	AWDM	Klebsiella pneumonia *	gentamycin	IZ 13.5 ± 0.34 mm MIC 12.5 µg/mL	[90]
	India	L	EtOH	AWDM	Pseudomonas aeruginosa *	gentamycin	IZ 10.9 ± 0.20 mm MIC 12.5 µg/mL	[90]
	India	L	EtOH	AWDM	Proteus vulgaris *	gentamycin	IZ 12.5 ± 0.28 mm MIC 6.25 µg/mL	[90]
	India	L	HX	DDM	*Staphylococcus aureus* MTCC 3160	methicillin	IZ 10.3 mm	[91]
	India	L	HX	DDM	*Bacillus subtilis* MTCC 619	methicillin	IZ 11.6 mm	[91]
	India	L	HX	DDM	*Escherichia coli* MTCC 4296	methicillin	IZ 10.6 mm	[91]
	India	L	HX	DDM	*Pseudomonas aeruginosa* MTCC 2488	methicillin	IZ 10.0 mm	[91]
	India	L	HX	DDM	*Candida albicans* MTCC 3018	methicillin	IZ 9.6 mm	[91]
	India	L	HX	DDM	*Pseudomonas aeruginosa* MTCC 2488	methicillin	IZ 10.0 mm	[91]
	India	L	HX	DDM	*Pseudomonas aeruginosa* MTCC 2488	methicillin	IZ 10.0 mm	[91]
	India	L	EtOAc	DDM	*Staphylococcus aureus* MTCC 3160	methicillin	IZ 11.6 mm	[91]
	India	L	EtOAc	DDM	*Bacillus subtilis* MTCC 619	methicillin	IZ 13.3 mm	[91]
	India	L	EtOAc	DDM	*Escherichia coli* MTCC 4296	methicillin	IZ 15.6 mm	[91]
	India	L	EtOAc	DDM	*Pseudomonas aeruginosa* MTCC 2488	methicillin	IZ 15.0 mm	[91]
	India	L	EtOAc	DDM	*Candida albicans* MTCC 3018	methicillin	IZ 15.3 mm	[91]
	India	L	MeOH	DDM	*Staphylococcus aureus* MTCC 3160	methicillin	IZ 21.6 mm	[91]
	India	L	MeOH	DDM	*Bacillus subtilis* MTCC 619	methicillin	IZ 19.6 mm	[91]
	India	L	MeOH	DDM	*Escherichia coli MTCC 4296*	methicillin	IZ 18.3 mm	[91]
	India	L	MeOH	DDM	*Pseudomonas aeruginosa* MTCC 2488	methicillin	IZ 18.6 mm	[91]
	India	L	MeOH	DDM	*Candida albicans* MTCC 3018	methicillin	IZ 17.6 mm	[91]
	India	L	MeOH	AWDM	Staphylococcus aureus *	na	IZ 14.0 mm	[92]
	India	B	MeOH	AWDM	Staphylococcus aureus *	na	IZ 8.6 mm	[92]
	India	L	MeOH	AWDM	Escherichia coli *	na	IZ 22.5 mm	[92]
	India	B	MeOH	AWDM	Escherichia coli *	na	IZ 14.13 mm	[92]
	India	L	MeOH	AWDM	Bacillus subtilis *	na	IZ 11.16 mm	[92]
	India	B	MeOH	AWDM	Bacillus subtilis *	na	IZ 6.23 mm	[92]
	India	L	MeOH	AWDM	Klebsiella pneumonia *	na	IZ 8.50 mm	[92]
	India	B	MeOH	AWDM	Klebsiella pneumonia *	na	IZ 4.4 mm	[92]
	India	L	MeOH	AWDM	Staphylococcus aureus *	na	IZ 14.1 mm	[92]
	India	B	MeOH	AWDM	Staphylococcus aureus *	na	IZ 8.8 mm	[92]
	India	L	MeOH	AWDM	Escherichia coli *	na	IZ 22.8 mm	[92]
	India	B	MeOH	AWDM	Escherichia coli *	na	IZ 14.22 mm	[92]
	India	L	MeOH	AWDM	Bacillus subtilis *	na	IZ 11.05 mm	[92]
	India	B	MeOH	AWDM	Bacillus subtilis *	na	IZ 6.8 mm	[92]
	India	L	MeOH	AWDM	Klebsiella pneumonia *	na	IZ 8.22 mm	[92]
	India	B	MeOH	AWDM	Klebsiella pneumonia *	na	IZ 4.26 mm	[92]
	India	L	Et2O	BMicDM	*Escherichia coli* MTCC-724	na	MIC 3.28 ± 1.24 μg/mL	[93]
	India	L	Et2O	BMicDM	*Enterobacter aerogenes* MTCC-39	na	MIC 21.26 ± 1.04 μg/mL	[93]
	India	L	Et2O	BMicDM	*Enterococcus faecalis* MTCC-2729	na	MIC 21.07 ± 1.70 μg/mL	[93]
	India	L	MeOH	BMicDM	*Escherichia coli* MTCC-724	na	MIC 3.28 ± 1.24 μg/mL	[93]
	India	L	MeOH	BMicDM	*Enterobacter aerogenes* MTCC-39	na	MIC 21.26 ± 1.04 μg/mL	[93]
	India	L	MeOH	BMicDM	*Enterococcus faecalis* MTCC-2729	na	MIC 21.07 ± 1.70 μg/mL	[93]
	Bangladesh	L	MeOH	BMicDM	*Staphylococcus aureus* BMLRU1002 and *pseudomonas aeruginosa* BMLRU1007	tetracycline	MIC 0.312 μg/mL	[94]
	Bangladesh	L	MeOH	BMicDM	*Bacillus subtilis* BMLRU1008 *Salmonella typhi* BMLRU1009	tetracycline	MIC 1.255 μg/mL	[94]
	Bangladesh	L	MeOH	BMicDM	*Escherichia coli BMLRU1001*	tetracycline	MIC 0.60 μg/mL	[94]
	India	L	EtOAc	DDM	Escherichia coli *	chloramphenicol	IZ 12 mm	[95]
	India	L	EtOAc	DDM	Klebsiella aerogenes *	chloramphenicol	IZ 13 mm	[95]
	India	L	EtOAc	DDM	Proteus vulgaris *	chloramphenicol	IZ 16 mm	[95]
	India	L	EtOAc	DDM	Pseudomonas aerogenes *	chloramphenicol	IZ 20 mm	[95]
	India	L	EtOAc	DDM	Escherichia coli *	chloramphenicol	IZ 14 mm	[95]
	India	L	EtOAc	DDM	Klebsiella aerogenes *	chloramphenicol	IZ 16 mm	[95]
	India	L	EtOAc	DDM	Proteus vulgaris *	chloramphenicol	IZ 14 mm	[95]
	India	L	EtOAc	DDM	Pseudomonas aerogenes *	chloramphenicol	IZ 15 mm	[95]
	India	L	Et_2_O	DDM	Escherichia coli *	chloramphenicol	IZ 22 mm	[95]
	India	L	Et_2_O	DDM	Klebsiella aerogenes *	chloramphenicol	IZ 22 mm	[95]
	India	L	Et_2_O	DDM	Proteus vulgaris *	chloramphenicol	IZ 19 mm	[95]
	India	L	Et_2_O	DDM	Pseudomonas aerogenes *	chloramphenicol	IZ 20 mm	[95]
	India	L	MeOH	DDM	Escherichia coli *	chloramphenicol	IZ 17 mm	[95]
	India	L	MeOH	DDM	Klebsiella aerogenes *	chloramphenicol	IZ 15 mm	[95]
	India	L	MeOH	DDM	Proteus vulgaris *	chloramphenicol	IZ 19 mm	[95]
	India	L	MeOH	DDM	Pseudomonas aerogenes *	chloramphenicol	IZ 19 mm	[95]
	Vietnam	L	EtOH	BDM	Escherichia coli and Staphylococcus aureus *	spiramycin	MIC 90 μg/mL	[96]
	India	L	MeOH	DDM	Pseudomonas aerogenes *	na	IZ 7 mm	[97]
	India	L	MeOH	DDM	Klebsiella pneumonia *	na	IZ 9 mm	[97]
	India	L	MeOH	DDM	Staphylococcus aureus *	na	IZ 7 mm	[97]
	India	L	MeOH	DDM	Bacillus cereus *	na	IZ 8 mm	[97]
	India	L	MeOH	BMacDM	*Bacillus cereus* NCIM 2155	ciprofloxacin	MIC 500 µg/mL MBC 1000 µg/mL IZ 13.550 ± 0.473 mm	[82]
	India	L	MeOH	BMacDM	*Bacillus pumilus* NCIM 2327	ciprofloxacin	MIC 500 µg/mL MBC 1000 µg/mL IZ 12.280 ± 0.710 mm	[82]
	India	L	MeOH	BMacDM	*Bacillus subtilis* NCIM 2063	ciprofloxacin	MIC 1000 µg/mL MBC 2000 µg/mL IZ 12.060 ± 0.877 mm	[82]
	India	L	MeOH	BMacDM	*Micrococcus luteus* NCIM 2376	ciprofloxacin	MIC 500 µg/mL MBC 1000 µg/mL IZ 13.180 ± 0.499 mm	[82]
	India	L	MeOH	BMacDM	*Staphylococcus aureus* NCIM 2901	ciprofloxacin	MIC 500 µg/mL MBC 1000 µg/mL IZ 13.410 ± 0.717 mm	[82]
	India	L	MeOH	BMacDM	*Escherichia coli* NCIM 2256	ciprofloxacin	MIC 2000 µg/mL MBC 4000 µg/mL IZ 7.920 ± 0.917 mm	[82]
	India	L	MeOH	BMacDM	*Klebsiella pneumoniae* NCIM 2957	ciprofloxacin	MIC 2000 µg/mL MBC 4000 µg/mL IZ 8.360 ± 0.439 mm	[82]
	India	L	MeOH	BMacDM	*Pseudomonas aeruginosa* NCIM 5031	ciprofloxacin	MIC 2000 µg/mL MBC 4000 µg/mL IZ 8.070 ± 0.767 mm	[82]
	India	L	MeOH	BMacDM	*Proteus vulgaris* NCIM 2027	ciprofloxacin	MIC 2000 µg/mL MBC 4000 µg/mL IZ 8.800 ± 0.822 mm	[82]
	India	L	MeOH	BMacDM	*Salmonella typhimurium* NCIM 2501	ciprofloxacin	MIC 2000 µg/mL MBC 4000 µg/mL IZ 7.430 ± 0.473 mm	[82]
	India	L	MeOH	BMacDM	*Shigella flexneri* MTCC 1457	ciprofloxacin	MIC 1000 µg/mL MBC 2000 µg/mL IZ 10.590 ± 0.452 mm	[82]
	India	L	MeOH	BMacDM	*Shigella sonnei* MTCC 2597	ciprofloxacin	MIC 1000 µg/mL MBC 2000 µg/mL IZ 11.380 ± 0.469 mm	[82]
	Bangladesh	L	MeOH	DDM	*Vibrio cholerae* AY-1868921	ciprofloxacin	IZ 21.133 ± 0.503 mm	[48]
	Bangladesh	L	MeOH	DDM	*Vibrio cholerae* O139 NIHCO270	ciprofloxacin	IZ 19.700 ± 0.529 mm	[48]
	Bangladesh	L	MeOH	DDM	*Escherichia coli* O157:H7 M-885496	ciprofloxacin	IZ 15.233 ± 0.351 mm	[48]
	Bangladesh	L	MeOH	DDM	*Shigella dysenteriae* Vm110432	ciprofloxacin	IZ 10.566 ± 0.568 mm	[48]
	Bangladesh	L	MeOH	DDM	*Shigella flexneri* M-12163	ciprofloxacin	IZ 12.066 ± 0.568 mm	[48]
	Bangladesh	L	MeOH	DDM	*Shigella boydi* M-297092	ciprofloxacin	IZ 11.733 ± 0.723 mm	[48]
	Bangladesh	L	MeOH	DDM	*Shigella sonnei* M-275521	ciprofloxacin	IZ 13.466 ± 0.288 mm	[48]
	Bangladesh	L	MeOH	DDM	*Vibrio parahaemolyticus* AQ-3794	ciprofloxacin	IZ 18.466 ± 0.472 mm	[48]
	Bangladesh	L	MeOH	DDM	*Vibrio mimicus* MGL-2585	ciprofloxacin	IZ 9.966 ± 0.702 mm	[48]
	Bangladesh	L	MeOH	DDM	*Aeromonas sobria* MGL-3585/1	ciprofloxacin	IZ 16.700 ± 0.435 mm	[48]
	Bangladesh	L	MeOH	DDM	Aeromonas cavie MGL-3615/1	ciprofloxacin	IZ 17.733 ± 0.568 mm	[48]
	Pakistan	L	MeOH	ADM	*Bacillus subtilis* ATCC 6633	erythromycin and cefixime	MIC 1µg/mL	[98]
	Pakistan	L	MeOH	ADM	*Enterococcus faecalis* ATCC 19433	erythromycin and cefixime	MIC 1 µg/mL	[98]
	Pakistan	L	MeOH	ADM	*Pseudomonas aeruginosa* ATCC 7221	erythromycin and cefixime	MIC 1 µg/mL	[98]
	Pakistan	L	MeOH	ADM	*Vibrio cholera* ATCC 11623	erythromycin and cefixime	MIC 1 µg/mL	[98]
	Pakistan	L	MeOH	ADM	*Entrobacter coccus* ATCC 13048	erythromycin and cefixime	MIC 1 µg/mL	[98]
	Pakistan	L	MeOH	ADM	*Klibsella pneumonia* ATCC UC57	erythromycin and cefixime	MIC 1 µg/mL	[98]
	Nepal	L	MeOH	AWPM	*Bacillus subtilis* ATCC6051	ampicillin	MBC 1.562 µg/mL	[49]
	Nepal	L	MeOH	AWPM	*Staphylococcus aureus* ATCC653P	ampicillin	MBC 6.25 µg/mL	[49]
	Nepal	L	MeOH	AWPM	*Bacillus subtilis* ATCC6051	ampicillin	MBC 2.372µg/mL	[49]
	Nepal	L	MeOH	AWPM	*Staphylococcus aureus* ATCC653P	ampicillin	MBC 0.245 µg/mL	[49]
	India	L	MeOH	BDM	*Escherichia coli* Dk1	methicillin	MIC 35.00 µg/mL	[99]
	India	L	MeOH	BDM	*Staphylococcus aureus* MRS901	methicillin	MIC 40.00 µg/mL	[99]
	India	R	MeOH and DCM	AWDM	Vibrio cholerae 3906	fluconazole and clotrimazole	IZ 12.73 ± 0.64 mm	[100]
	India	R	MeOH and DCM	AWDM	*Escherichia coli* 118	fluconazole and clotrimazole	IZ 21.9 ± 0.85 mm	[100]
	India	R	MeOH and DCM	AWDM	*Escherichia coli* 614	fluconazole and clotrimazole	IZ 18.8 ± 0.72 mm	[100]
	India	R	MeOH and DCM	AWDM	*Shigella flexneri* 1457	fluconazole and clotrimazole	IZ 12.3 ± 1.2 mm	[100]
	India	R	MeOH and DCM	AWDM	*Shigella flexneri* 9543	fluconazole and clotrimazole	IZ 17.16 ± 1.04 mm	[100]
	India	R	MeOH and DCM	AWDM	*Salmonella enterica typhimurium* 98	fluconazole and clotrimazole	IZ 15.8 ± 0.72 mm	[100]
	India	R	MeOH and DCM	AWDM	*Salmonella enterica ser. typhi* 733	fluconazole and clotrimazole	IZ 10.8 ± 0.08 mm	[100]
	India	R	MeOH and DCM	AWDM	*Salmonella paratyphi* 3220	fluconazole and clotrimazole	IZ 11.43 ± 0.4 mm	[100]
	India	R	MeOH and DCM	AWDM	*Klebsiella pneumoniae* 109	fluconazole and clotrimazole	IZ 18.4 ± 0.64 mm	[100]
	India	R	MeOH and DCM	AWDM	*Pseudomonas aeruginosa* 1035	fluconazole and clotrimazole	IZ 12.1 ± 1.7 mm	[100]
	India	R	MeOH and DCM	AWDM	*Pseudomonas aeruginosa* 1035	fluconazole and clotrimazole	IZ 12.1 ± 1.7 mm	[100]
	India	R	MeOH and DCM	AWDM	*Enterococcus faecalis* 2729	fluconazole and clotrimazole	IZ 13.6 ± 1.23 mm	[100]
	India	R	MeOH and DCM	AWDM	*Staphylococcus aureus* 1430	fluconazole and clotrimazole	IZ 14.7 ± 0.82 mm	[100]
	India	L	EtOH	PDM	*Bacillus subtilis* ATCC6633	amoxicillin	IZ 0.21 mm	[101]
	India	L	PE	PDM	*Bacillus subtilis* ATCC6633	amoxicillin	IZ 0.21 mm	[101]
	India	L	EtOH	PDM	*Bacillus subtilis* ATCC6633	amoxicillin	IZ 0.25 mm	[101]
	India	L	PE	PDM	*Bacillus subtilis* ATCC6633	amoxicillin	IZ 0.25 mm	[101]
	India	L	EtOH	PDM	*Staphylococcus aureus* ATCC6538P	amoxicillin	IZ 0.25 mm	[101]
	India	L	PE	PDM	*Staphylococcus aureus* ATCC6538P	amoxicillin	IZ 0.21 mm	[101]
	India	L	EtOH	PDM	*Staphylococcus aureus* ATCC6538P	amoxicillin	IZ 0.21 mm	[101]
	India	L	PE	PDM	*Staphylococcus aureus* ATCC6538P	amoxicillin	IZ 0.25 mm	[101]
	India	L	EtOH	DDM	*Staphylococcus aureus* ATCC6538P	amoxicillin	IZ 0.34 ± 0.06 mm	[101]
	India	L	PE	DDM	*Staphylococcus aureus* ATCC6538P	amoxicillin	IZ 0.53 ± 0.07 mm	[101]
	India	L	EtOH	DDM	*Staphylococcus aureus* ATCC6538P	amoxicillin	IZ 0.53 ± 0.09 mm	[101]
	India	L	PE	DDM	*Bacillus subtilis* ATCC6633	amoxicillin	IZ 0.42 ± 0.13 mm	[101]
	India	L	EtOH	DDM	*Bacillus subtilis* ATCC6633	amoxicillin	IZ 0.46 ± 0.06 mm	[101]
	India	L	PE	DDM	*Bacillus subtilis* ATCC6633	amoxicillin	IZ 0.5 ± 0.08 mm	[101]
	India	Sb	HX	AWDM	*Escherichia coli* MTCC B1560	ciprofloxacin	MIC > 1000 µg/mL	[102]
	India	Sb	Chl	AWDM	*Escherichia coli* MTCC B1560	ciprofloxacin	MIC > 1000 µg/mL	[102]
	India	Sb	MeOH	AWDM	*Escherichia coli* MTCC B1560	ciprofloxacin	MIC 500 µg/mL	[102]
	India	Sb	HX	AWDM	*Klebsiella pneumoniae* MTCC B4030	ciprofloxacin	MIC 1000 µg/mL	[102]
	India	Sb	Chl	AWDM	*Klebsiella pneumoniae* MTCC B4030	ciprofloxacin	MIC 1000 µg/mL	[102]
	India	Sb	MeOH	AWDM	*Klebsiella pneumoniae* MTCC B4030	ciprofloxacin	MIC 250 µg/mL	[102]
	India	Sb	HX	AWDM	*Pseudomonas aeruginosa* MTCC B2297	ciprofloxacin	MIC 1000 µg/mL	[102]
	India	Sb	Chl	AWDM	*Pseudomonas aeruginosa* MTCC B2297	ciprofloxacin	MIC 250 µg/mL	[102]
	India	Sb	MeOH	AWDM	*Pseudomonas aeruginosa* MTCC B2297	ciprofloxacin	MIC 62.5 µg/mL	[102]
	India	Sb	HX	AWDM	*Proteus vulgaris* MTCC B7299	ciprofloxacin	MIC 500 µg/mL	[102]
	India	Sb	Chl	AWDM	*Proteus vulgaris* MTCC B7299	ciprofloxacin	MIC 62.5 µg/mL	[102]
	India	Sb	MeOH	AWDM	*Proteus vulgaris* MTCC B7299	ciprofloxacin	MIC 31.2 µg/mL	[102]
	India	Sb	HX	AWDM	*Bacillus subtilis* MTCC B2274	ciprofloxacin	MIC > 1000 µg/mL	[102]
	India	Sb	Chl	AWDM	*Bacillus subtilis* MTCC B2274	ciprofloxacin	MIC 1000 µg/mL	[102]
	India	Sb	MeOH	AWDM	*Bacillus subtilis* MTCC B2274	ciprofloxacin	MIC 500 µg/mL	[102]
	India	Sb	HX	AWDM	*Enterococcus faecalis* MTCC B3159	ciprofloxacin	MIC 1000 µg/mL	[102]
	India	Sb	Chl	AWDM	*Enterococcus faecalis* MTCC B3159	ciprofloxacin	MIC 1000 µg/mL	[102]
	India	Sb	MeOH	AWDM	*Enterococcus faecalis* MTCC B3159	ciprofloxacin	MIC 250 µg/mL	[102]
	India	Sb	HX	AWDM	*Micrococcus luteus* MTCC B1538	ciprofloxacin	MIC > 1000 µg/mL	[102]
	India	Sb	Chl	AWDM	*Micrococcus luteus* MTCC B1538	ciprofloxacin	MIC 500 µg/mL	[102]
	India	Sb	MeOH	AWDM	*Micrococcus luteus* MTCC B1538	ciprofloxacin	MIC 62.5 µg/mL	[102]
	India	Sb	HX	AWDM	*Staphylococcus aureus* MTCC B3160	ciprofloxacin	MIC > 1000 µg/mL	[102]
	India	Sb	Chl	AWDM	*Staphylococcus aureus* MTCC B3160	ciprofloxacin	MIC 500 µg/mL	[102]
	India	Sb	MeOH	AWDM	*Staphylococcus aureus* MTCC B3160	ciprofloxacin	MIC 250 µg/mL	[102]
	India	Sb	HX	AWDM	*Streptococcus pneumoniae* MTCC B2672	ciprofloxacin	MIC 1000 µg/mL	[102]
	India	Sb	Chl	AWDM	*Streptococcus pneumoniae* MTCC B2672	ciprofloxacin	MIC 500 µg/mL	[102]
	India	Sb	MeOH	AWDM	*Streptococcus pneumoniae* MTCC B2672	ciprofloxacin	MIC 62.5 µg/mL	[102]
	India	L	MeOH	AWDM	*Staphylococcus aureus* MTCC 1144	gentamicin and ciprofloxacin	MIC 5000 µg/mL	[103]
	India	L	MeOH	AWDM	Escherichia coli *	gentamicin and ciprofloxacin	MIC 2500 µg/mL	[103]
	India	L	MeOH	AWDM	Shigella flexneri *	gentamicin and ciprofloxacin	MIC 1250 µg/mL	[103]
	India	L	MeOH	AWDM	*Vibrio cholerae* MTCC 3904	gentamicin and ciprofloxacin	MIC 5000 µg/mL	[103]
	India	B	MeOH	AWDM	*Staphylococcus aureus* MTCC 1144	gentamicin and ciprofloxacin	MIC 5000 µg/mL	[103]
	India	L	MeOH	AWDM	Escherichia coli *	gentamicin and ciprofloxacin	MIC 5000 µg/mL	[103]
	India	L	MeOH	AWDM	Shigella flexneri*	gentamicin and ciprofloxacin	MIC 5000 µg/mL	[103]
	India	L	MeOH	AWDM	*Vibrio cholerae* MTCC 3904	gentamicin and ciprofloxacin	MIC 5000 µg/mL	[103]
	India	B	PE	DDM	*Bacillus subtilis* MTCC 7164	ampicillin	IZ 10.3 ± 1.15 mm	[104]
	India	B	PE	DDM	*Staphylococcus aureus* MTCC 1144	ampicillin	IZ 11.6 ± 0.57 mm	[104]
	India	B	PE	DDM	*Escherichia coli* MTCC 1098	ampicillin	IZ 12.6 ± 0.57 mm	[104]
	India	B	PE	DDM	*Pseudomonas aeruginosa* MTCC 1034	ampicillin	IZ 11.0 ± 0.00 mm	[104]
	India	B	PE	DDM	*Vibrio cholerae* MTCC 3904	ampicillin	IZ 11.0 ± 0.00 mm	[104]
	India	B	PE	DDM	*V. alginolyteus* MTCC 4439	ampicillin	IZ 12.6 ± 0.57 mm	[104]
	India	L	PE	DDM	*Bacillus subtilis* MTCC 7164	ampicillin	IZ 8.6 ± 0.57 mm	[104]
	India	L	PE	DDM	*Staphylococcus epidermidis* MTCC 3615	ampicillin	IZ 11.3 ± 0.57 mm	[104]
	India	L	PE	DDM	*Escherichia coli* MTCC 1098	ampicillin	IZ 12.3 ± 0.57 mm	[104]
	India	L	PE	DDM	*Salmonella typhimurium* MTCC 3216	ampicillin	IZ 10.0 ± 1.73 mm	[104]
	India	L	PE	DDM	*Pseudomonas aeruginosa* MTCC 1034	ampicillin	IZ 11.0 ± 0.00 mm	[104]
	India	L	PE	DDM	*Vibrio cholerae* MTCC 3904	ampicillin	IZ 11.0 ± 0.00 mm	[104]
	India	L	PE	DDM	*V. alginolyteus* MTCC 4439	ampicillin	IZ 13.0 ± 0.57 mm	[104]
	India	B	Chl	DDM	*Bacillus subtilis* MTCC 7164	ampicillin	IZ 9.6 ± 2.08 mm	[104]
	India	B	Chl	DDM	*Staphylococcus aureus* MTCC 1144	ampicillin	IZ 10.3 ± 0.57 mm	[104]
	India	B	Chl	DDM	*Staphylococcus epidermidis* MTCC 3615	ampicillin	IZ 13.6 ± 0.57 mm	[104]
	India	B	Chl	DDM	*Escherichia coli* MTCC 1098	ampicillin	IZ 13.6 ± 0.57 mm	[104]
	India	B	Chl	DDM	*Pseudomonas aeruginosa* MTCC 1034	ampicillin	IZ 10.6 ± 0.57 mm	[104]
	India	B	Chl	DDM	*Vibrio cholerae* MTCC 3904	ampicillin	IZ 9.6 ± 0.57 mm	[104]
	India	B	Chl	DDM	*V. alginolyteus* MTCC 4439	ampicillin	IZ 12.3 ± 1.52 mm	[104]
	India	L	Chl	DDM	*Bacillus subtilis* MTCC 7164	ampicillin	IZ 10.3 ± 0.57 mm	[104]
	India	L	Chl	DDM	*Staphylococcus epidermidis* MTCC 3615	ampicillin	IZ 11.6 ± 0.57 mm	[104]
	India	L	Chl	DDM	*Escherichia coli* MTCC 1098	ampicillin	IZ 13.0 ± 1.00 mm	[104]
	India	L	Chl	DDM	*Pseudomonas aeruginosa* MTCC 1034	ampicillin	IZ 12.3 ± 0.57 mm	[104]
	India	L	Chl	DDM	*Vibrio cholerae* MTCC 3904	ampicillin	IZ 11.6 ± 0.57 mm	[104]
	India	L	Chl	DDM	*V. alginolyteus* MTCC 4439	ampicillin	IZ 11.6 ± 0.57 mm	[104]
	India	B	EtOH	DDM	*Bacillus subtilis* MTCC 7164	ampicillin	IZ 11. 6 ± 0.57 mm	[104]
	India	B	EtOH	DDM	*Staphylococcus epidermidis* MTCC 3615	ampicillin	IZ 11.6 ± 0.57 mm	[104]
	India	B	EtOH	DDM	*Escherichia coli* MTCC 1098	ampicillin	IZ 13.6 ± 0.57 mm	[104]
	India	B	EtOH	DDM	*Salmonella typhimurium* MTCC 3216	ampicillin	IZ 11.3 ± 0.57mm	[104]
	India	B	EtOH	DDM	*Pseudomonas aeruginosa* MTCC 1034	ampicillin	IZ 13.0 ± 0.00 mm	[104]
	India	B	EtOH	DDM	*Vibrio cholerae* MTCC 3904	ampicillin	IZ 11.6 ± 0.57 mm	[104]
	India	B	EtOH	DDM	*V. alginolyteus* MTCC 4439	ampicillin	IZ 11.6 ± 0.57 mm	[104]
	India	L	EtOH	DDM	*Bacillus subtilis* MTCC 7164	ampicillin	IZ 11.6 ± 0.57 mm	[104]
	India	L	EtOH	DDM	*Staphylococcus epidermidis* MTCC 3615	ampicillin	IZ 13.6 ± 0.57 mm	[104]
	India	L	EtOH	DDM	*Escherichia coli MTCC 1098*	ampicillin	IZ 16.3 ± 1.52 mm	[104]
	India	L	EtOH	DDM	*Salmonella typhimurium* MTCC 3216	ampicillin	IZ 11.3 ± 0.57 mm	[104]
	India	L	EtOH	DDM	*Pseudomonas aeruginosa* MTCC 1034	ampicillin	IZ 11.6 ± 0.57 mm	[104]
	India	L	EtOH	DDM	*Vibrio cholerae* MTCC 3904	ampicillin	IZ 9.6 ± 0.57 mm	[104]
	India	L	EtOH	DDM	*V. alginolyteus* MTCC 4439	ampicillin	IZ 11.6 ± 0.57 mm	[104]
	India	B	MeOH	DDM	*Bacillus subtilis* MTCC 7164	ampicillin	IZ 13.0 ± 1.00 mm	[104]
	India	B	MeOH	DDM	*Staphylococcus aureus* MTCC 1144	ampicillin	IZ 11.3 ± 1.15 mm	[104]
	India	B	MeOH	DDM	*Staphylococcus epidermidis* MTCC 3615	ampicillin	IZ 13.6 ± 0.57 mm	[104]
	India	B	MeOH	DDM	*Escherichia coli* MTCC 1098	ampicillin	IZ 12.3 ± 0.57 mm	[104]
	India	B	MeOH	DDM	*Salmonella typhimurium* MTCC 3216	ampicillin	IZ 11.6 ± 0.57 mm	[104]
	India	B	MeOH	DDM	*Pseudomonas aeruginosa* MTCC 1034	ampicillin	IZ 10.6 ± 1.15 mm	[104]
	India	B	MeOH	DDM	*Vibrio cholerae* MTCC 3904	ampicillin	IZ 13.6 ± 0.57 mm	[104]
	India	B	MeOH	DDM	*V. alginolyteus* MTCC 4439	ampicillin	IZ 9.6 ± 0.57 mm	[104]
	India	L	MeOH	DDM	*Bacillus subtilis* MTCC 7164	ampicillin	IZ 9.3 ± 0.57 mm	[104]
	India	L	MeOH	DDM	*Staphylococcus aureus* MTCC 1144	ampicillin	IZ 8.0 ± 0.00 mm	[104]
	India	L	MeOH	DDM	*Staphylococcus epidermidis* MTCC 3615	ampicillin	IZ 11.3 ± 1.15 mm	[104]
	India	L	MeOH	DDM	*Escherichia coli* MTCC 1098	ampicillin	IZ 12.3 ± 0.57 mm	[104]
	India	L	MeOH	DDM	*Salmonella typhimurium* MTCC 3216	ampicillin	IZ 9.0 ± 1.00 mm	[104]
	India	L	MeOH	DDM	*Pseudomonas aeruginosa* MTCC 1034	ampicillin	IZ 13.3 ± 0.57 mm	[104]
	India	L	MeOH	DDM	*Vibrio cholerae* MTCC 3904	ampicillin	IZ 11.6 ± 0.57 mm	[104]
	India	L	MeOH	DDM	*V. alginolyteus* MTCC 4439	ampicillin	IZ 9.6 ± 0.57 mm	[104]
	India	L	na	na	Staphylococcus aureus *	na	IZ 14 mm	[105]
	India	L	na	na	Proteus mirabilis *	na	IZ 10 mm	[105]
	India	L	na	na	Vibrio cholerae *	na	IZ 12 mm	[105]
	India	L	na	na	Pseudomonas aeruginosa *	na	IZ 12 mm	[105]
	India	L	MeOH	AWDM	*Klebsiella pneumoniae* MTCC 7407	rifampicin	IZ 13.0 ± 0.21 mm	[106]
	India	L	MeOH	AWDM	*Staphylococc usaureus* MTCC 96	rifampicin	IZ 11.0 ± 0.12 mm	[106]
	India	L	EtOH	BMicDM	Streptococcus faecalis *	fluconazole	MIC 125 µg/mL	[107]
	India	L	EtOH	BMicDM	Klebsiella pneumoniae *	fluconazole	MIC 250 µg/mL	[107]
	India	L	EtOH	BMicDM	Escherichia coli *	fluconazole	MIC 250 µg/mL	[107]
	India	L	EtOH	BMicDM	Pseudomonas aeruginosa *	fluconazole	MIC 500 µg/mL	[107]
	India	L	EtOH	BMicDM	Staphylococcus aureus *	fluconazole	MIC 250 µg/mL	[107]
	India	L	EtOH	DDM	*Bacillus cereus* NCIM 2156	chloramphenicol	IZ 0.68 mm	[108]
	India	L	EtOH	DDM	*Staphylococcus aureus* NCIM 2654	chloramphenicol	IZ 0.66 mm	[108]
	India	L	EtOH	DDM	*S. epidermidis* NCIM 2493	chloramphenicol	IZ 0.71 mm	[108]
	India	L	EtOH	DDM	*Mycobacterium smegmatis* NCIM 5138	chloramphenicol	IZ 0.88 mm	[108]
	India	Se	EtOH	DDM	*Bacillus cereus* NCIM 2156	chloramphenicol	IZ 0.72 mm	[108]
	India	Se	EtOH	DDM	*Staphylococcus aureus* NCIM 2654	chloramphenicol	IZ 0.63 mm	[108]
	India	Se	EtOH	DDM	*S. epidermidis* NCIM 2493	chloramphenicol	IZ 0.76 mm	[108]
	India	Se	EtOH	DDM	*Mycobacterium smegmatis* NCIM 5138	chloramphenicol	IZ 0.72 mm	[108]
	India	L	EtOH	DDM	*Escherichia coli* NCIM 2027	streptomycin	IZ 0.88 mm	[108]
	India	L	EtOH	DDM	*Pseudomonas aeruginosa* NCIM 5032	streptomycin	IZ 1.0 mm	[108]
	India	L	EtOH	DDM	*Proteus vulgaris* NCIM 2027	streptomycin	IZ 0.78 mm	[108]
	India	L	EtOH	DDM	*Salmonella typhimurium* NCIM 2501	streptomycin	IZ 0.80 mm	[108]
	India	Se	EtOH	DDM	*Escherichia coli* NCIM 2027	streptomycin	IZ 0.86 mm	[108]
	India	Se	EtOH	DDM	*Pseudomonas aeruginosa* NCIM 5032	streptomycin	IZ 0.90 mm	[108]
	India	Se	EtOH	DDM	*Proteus vulgaris* NCIM 2027	streptomycin	IZ 0.84 mm	[108]
	India	Se	EtOH	DDM	*Salmonella typhimurium* NCIM 2501	streptomycin	IZ 0.72 mm	[108]
	India	L	EtOH	BDM	*Staphylococcus aureus* MTCC 7443	chloramphenicol	MIC 2000 µg/mL MBC 4000 µg/mL	[109]
	India	L	EtOH	BDM	*Micrococcus luteus* MTCC 4821	chloramphenicol	MIC 2000 µg/mL MBC 4000 µg/mL	[109]
	India	L	EtOH	BDM	*Bacillus subtilis* MTCC 2389	chloramphenicol	MIC 2000 µg/mL MBC 4000 µg/mL	[109]
	India	L	EtOH	BDM	*Escherichia coli* MTCC 2127	ampicillin	MIC 2000 µg/mL MBC 4000 µg/mL	[109]
	India	L	EtOH	BDM	*Klebsiella pneumoniae* MTCC 7172	ampicillin	MIC 2000 µg/mL MBC 4000 µg/mL	[109]
	India	L	na	DDM	Klebsiella pneumoniae *	na	MIC 400 µg/mL	[110]
	India	L	na	DDM	Escherichia coli *	na	MIC 400 µg/mL	[110]
	India	L	na	DDM	*Salmonella Para typh*	na	MIC 400 µg/mL	[110]
	India	L	na	DDM	Salmonella typhi *	na	MIC 400 µg/mL	[110]
	Malaysia	L	MeOH	DDM	Escherichia coli *	cefotaxime	IZ 28.0 ± 0.5 mm	[111]
	Malaysia	L	MeOH	DDM	Staphylococcus aureus *	cefotaxime	IZ 21.0 ± 1.5 mm	[111]
*V. obovata*	South Africa	L	MeOH	ADM	*Staphylococcus aureus* ATCC 6538	ciprofloxacin	MIC 0.02 µg/mL	[50]
	South Africa	L	MeOH	ADM	*Bacillus cereus* ATCC 11778	ciprofloxacin	MIC 0.02 µg/mL	[50]
	South Africa	L	MeOH	ADM	*Escherichia coli* ATCC 11775	ciprofloxacin	MIC 4.00 µg/mL	[50]
*V. peduncularis*	India	L	MeOH	BMacDM	*Bacillus cereus* NCIM 2155	ciprofloxacin	MIC 125 µg/mL MBC 250 µg/mL IZ 18.040 ± 0.876 mm	[82]
	India	L	MeOH	BMacDM	*Bacillus pumilus* NCIM 2327	ciprofloxacin	MIC 125 µg/mL MBC 1000 µg/mL IZ 16.770 ± 0.473 mm	[82]
	India	L	MeOH	BMacDM	*Bacillus subtilis* NCIM 2063	ciprofloxacin	MIC 125 µg/mL MBC 250 µg/mL IZ 17.160 ± 0.817mm	[82]
	India	L	MeOH	BMacDM	*Micrococcus luteus* NCIM 2376	ciprofloxacin	MIC 62.05 µg/mL MBC 125.0 µg/mL IZ 21.590 ± 0.821 mm	[82]
	India	L	MeOH	BMacDM	*Staphylococcus aureus* NCIM 2901	ciprofloxacin	MIC 62.05 µg/mL MBC 125.0 µg/mL IZ 22.600 ± 0.755 mm	[82]
	India	L	MeOH	BMacDM	*Escherichia coli NCIM 2256*	ciprofloxacin	MIC 500 µg/mL MBC 1000 µg/mL IZ 13.680 ± 0.520 mm	[82]
	India	L	MeOH	BMacDM	*Klebsiella pneumoniae* NCIM 2957	ciprofloxacin	MIC 1000 µg/mL MBC 2000 µg/mL IZ 11.190 ± 0.810 mm	[82]
	India	L	MeOH	BMacDM	*Pseudomonas aeruginosa* NCIM 5031	ciprofloxacin	MIC 500 µg/mL MBC 1000 µg/mL IZ 12.730 ± 0.452 mm	[82]
	India	L	MeOH	BMacDM	*Proteus vulgaris* NCIM 2027	ciprofloxacin	MIC 500 µg/mL MBC 1000 µg/mL IZ 12.430 ± 0.473 mm	[82]
	India	L	MeOH	BMacDM	*Salmonella typhimurium* NCIM 2501	ciprofloxacin	MIC 500 µg/mL MBC 1000 µg/mL IZ 12.590 ± 0.821 mm	[82]
	India	L	MeOH	BMacDM	*Shigella flexneri* MTCC 1457	ciprofloxacin	MIC 250 µg/mL MBC 500 µg/mL IZ 14.430 ± 0.391 mm	[82]
	India	L	MeOH	BMacDM	*Shigella sonnei* MTCC 2597	ciprofloxacin	MIC 250 µg/mL MBC 500 µg/mL IZ 15.310 ± 0.605 mm	[82]
*V. pinnata*	Indonesia	L	MeOH	BMicDM	Streptococcus mutans *	na	−1.48 μg/mL	[112]
	Indonesia	L	HX	BMicDM	Streptococcus mutans *	na	−1.45 μg/mL	[112]
	Indonesia	L	EtOAc	BMicDM	Streptococcus mutans *	na	−0.17 μg/mL	[112]
	Indonesia	B	EtOH	BDM	*Propionibacterium acnes* ATCC 6919	chloramphenicol	MIC 1.00 μg/mL	[54]
	Indonesia	B	MeOH	BDM	*Propionibacterium acnes* ATCC 6919	chloramphenicol	MIC 2.00 μg/mL	[54]
	Indonesia	S	EtOH	BDM	*Propionibacterium acnes* ATCC 6919	chloramphenicol	MIC 1.00 μg/mL	[54]
	Indonesia	S	MeOH	BDM	*Propionibacterium acnes* ATCC 6919	chloramphenicol	MIC 1.00 μg/mL	[54]
	Brunei	L	EtOAc	DDM	*Staphylococcus aureus* ATCC 29213	streptomycin	IZ 6.2 ± 0.5 mm	[113]
	Brunei	L	EtOAc	DDM	*Bacillus subtilis* ATCC 11774	streptomycin	IZ 9.3 ± 0.5 mm	[113]
	Brunei	L	Chl	DDM	*Bacillus subtilis* ATCC 11774	streptomycin	IZ 9.3 ± 0.1 mm	[113]
	Brunei	L	HX	DDM	*Bacillus subtilis* ATCC 11774	streptomycin	IZ 8.2 ± 1.2 mm	[113]
	Brunei	L	Chl	DDM	*Escherichia coli* ATCC 11775	streptomycin	IZ 8.4 ± 0.1 mm	[113]
	Brunei	L	EtOAc	DDM	*Escherichia coli* ATCC 11775	streptomycin	IZ 10.2 ± 0.3 mm	[113]
	Brunei	L	EtOAc	DDM	*Pseudomonas aeruginosa* ATCC 27853	streptomycin	IZ 6.2 ± 0.5 mm	[113]
*V. pooara*	South Africa	L	Ace	ADM	*Staphylococcus aureus* ATCC 6538	nordihydroguaiaretic	MIC 32 µg/mL	[114]
	South Africa	L	Ace	ADM	*Bacillus cereus* ATCC 11778	nordihydroguaiaretic	MIC 16 µg/mL	[114]
	South Africa	L	MeOH	ADM	*Staphylococcus aureus* ATCC 6538	ciprofloxacin	MIC 1.00 µg/mL	[50]
	South Africa	L	MeOH	ADM	*Bacillus cereus* ATCC 11778	ciprofloxacin	MIC 0.50 µg/mL	[50]
	South Africa	L	MeOH	ADM	*Escherichia coli* ATCC 11775	ciprofloxacin	MIC 8.00 µg/mL	[50]
*V. pseudo-negundo*	Iran	L	MeOH	AWDM	*Staphylococcus aureus* ATCC 25923	na	MIC 22.6 ± 0.3 µg/mL	[115]
	Iran	L	MeOH	AWDM	*Escherichia coli ATCC 35150*	na	MIC 17.1 ± 0.2 µg/mL	[115]
*V. rehmannii*	South Africa	L	Ace	ADM	*Staphylococcus aureus* ATCC 6538	nordihydroguaiaretic	MIC 16 µg/mL	[114]
	South Africa	L	Ace	ADM	*Bacillus cereusATCC 11778*	nordihydroguaiaretic	MIC 8 µg/mL	[114]
	South Africa	L	MeOH	ADM	*Staphylococcus aureus* ATCC 6538	nordihydroguaiaretic	MIC 0.02 µg/mL	[114]
	South Africa	L	MeOH	ADM	*Bacillus cereusATCC 11778*	nordihydroguaiaretic	MIC 0.02 µg/mL	[114]
	South Africa	L	MeOH	ADM	*Escherichia coli ATCC 11775*	nordihydroguaiaretic	MIC 4.00 µg/mL	[114]
*V. trifolia*	India	L	Peth	DDM	Pseudomonas aeruginosa *	chloramphenicol	IZ 5 mm	[116]
	India	L	Chl	DDM	Pseudomonas aeruginosa *	chloramphenicol	IZ 22 mm	[116]
	India	L	MeOH	DDM	Pseudomonas aeruginosa *	chloramphenicol	IZ 17 mm	[116]
	India	L	PE	DDM	Klebsiella pneumoniae *	chloramphenicol	IZ 18 mm	[116]
	India	L	Chl	DDM	Klebsiella pneumoniae *	chloramphenicol	IZ 22 mm	[116]
	India	L	MeOH	DDM	Klebsiella pneumoniae *	chloramphenicol	IZ 18 mm	[116]
	India	L	PE	DDM	Streptococcus pyogenes *	chloramphenicol	IZ 18 mm	[116]
	India	L	Chl	DDM	Streptococcus pyogenes *	chloramphenicol	IZ 20 mm	[116]
	India	L	MeOH	DDM	Streptococcus pyogenes *	chloramphenicol	IZ 17 mm	[116]
	India	L	PE	DDM	Staphylococcus aureus *	chloramphenicol	IZ 14 mm	[116]
	India	L	Chl	DDM	Staphylococcus aureus *	chloramphenicol	IZ 19 mm	[116]
	India	L	MeOH	DDM	Staphylococcus aureus *	chloramphenicol	IZ 15 mm	[116]
	India	L	MeOH	BMacDM	*Bacillus cereus* NCIM 2155	ciprofloxacin	MIC 250 µg/mL MBC 500 µg/mL IZ 15.490 ± 0.526 mm	[82]
	India	L	MeOH	BMacDM	*Bacillus pumilus* NCIM 2327	ciprofloxacin	MIC 500 µg/mL MBC 1000 µg/mL IZ 14.820 ± 0.320 mm	[82]
	India	L	MeOH	BMacDM	*Bacillus subtilis* NCIM 2063	ciprofloxacin	MIC 250 µg/mL MBC 500 µg/mL IZ 14.300 ± 0.611 mm	[82]
	India	L	MeOH	BMacDM	*Micrococcus luteus* NCIM 2376	ciprofloxacin	MIC 125 µg/mL MBC 250 µg/mL IZ 16.590 ± 0.452 mm	[82]
	India	L	MeOH	BMacDM	*Staphylococcus aureus* NCIM 2901	ciprofloxacin	MIC 125 µg/mL MBC 250 µg/mL IZ 17.500 ± 0.347 mm	[82]
	India	L	MeOH	BMacDM	*Escherichia coli* NCIM 2256	ciprofloxacin	MIC 1000 µg/mL MBC 2000 µg/mL IZ 11.550 ± 0.195 mm	[82]
	India	L	MeOH	BMacDM	*Klebsiella pneumoniae* NCIM 2957	ciprofloxacin	MIC 1000 µg/mL MBC 2000 µg/mL IZ 10.590 ± 0.821 mm	[82]
	India	L	MeOH	BMacDM	*Pseudomonas aeruginosa* NCIM 5031	ciprofloxacin	MIC 1000 µg/mL MBC 2000 µg/mL IZ 8.810 ± 0.790 mm	[82]
	India	L	MeOH	BMacDM	*Proteus vulgaris NCIM 2027*	ciprofloxacin	MIC 2000 µg/mL MBC 4000 µg/mL IZ 9.810 ± 0.330 mm	[82]
	India	L	MeOH	BMacDM	*Salmonella typhimurium* NCIM 2501	ciprofloxacin	MIC 2000 µg/mL MBC 4000 µg/mL IZ 10.420 ± 0.412 mm	[82]
	India	L	MeOH	BMacDM	*Shigella flexneri MTCC 1457*	ciprofloxacin	MIC 500 µg/mL MBC 1000 µg/mL IZ 12.250 ± 0.452 mm	[82]
	India	L	MeOH	BMacDM	*Shigella sonnei* MTCC 2597	ciprofloxacin	MIC 500 µg/mL MBC 1000 µg/mL IZ 12.930 ± 0.713 mm	[82]
	Malaysia	L	MeOH	DDM	*Bacillus ceres* NRRL 14591B	nystatin and streptomycin	MIC 62 µg/mL	[117]
	Malaysia	L	MeOH	DDM	*Pseudomonas aeruginosa* UI-60690	nystatin and streptomycin	MIC 125 µg/mL	[117]

Ace—Acetone; ADM—Agar Dilution Method; ADM—Agar Dilution Method; AWDM—Agar Well Dilution Method; B—Bark; BDM—Broth Dilution Method; BMacDM—Broth Macrodilution Method; BMicDM—Broth Microdilution Method; Chl—Chloroform; DCM—Dichloromethane; DDM—Disc Diffusion Method; EC_50_—Median Inhibition Concentration; Et_2_O—Diethyl Ether; EtoAc—Ethyle acetate; EtOH—Ethanol; F—Fruit; Fl—Flower; H_2_O—Water; HX—Hexane; IZ—Inhibition Zone; L—Leaf; MBC—Minimum Bactericidal Concentration; Mean ± standard error; MeOH—Methanol; MIC—Minimum Inhibition Concentration; na—Not available; PDM—Paper Disc Method; PE—Petroliumether; R—Root; Sb—Steambark; Se—Seed; *—Strain not indicated.

**Table 3:** *In vitro* antifungal activity studies on *Vitex* species.

Species	Country	Plant Part Use	Extractive Solvent	Test Type	Strains/Microorganism	Result/MIC/MFC (µg/mL, mm)	Positive Control	BR
*V. agnus castus*	Saudi Arabia	L	EtOH	AWDM	Candida tropicalis *	MFC 25 MIC 25	Nystatin (10 µg)	[118]
	Saudi Arabia	L	MeOH	AWDM	Candida tropicalis *	MFC 50 MIC 25	Nystatin (10 µg)	[118]
	Saudi Arabia	L	H_2_O	AWDM	Candida tropicalis *	MFC 50 MIC 25	Nystatin (10 µg)	[118]
	Saudi Arabia	L	EtOH	AWDM	Candida albicans *	MFC 50 MIC 25	Nystatin (10 µg)	[118]
	Saudi Arabia	L	MeOH	AWDM	Candida albicans *	MFC 100 MIC 25	Nystatin (10 µg)	[118]
	Saudi Arabia	L	H_2_O	AWDM	Candida albicans *	MFC 50 MIC 25	Nystatin (10 µg)	[118]
	Saudi Arabia	L	EtOH	AWDM	Candida ciferrii *	MFC 50 MIC 25	Nystatin (10 µg)	[118]
	Saudi Arabia	L	MeOH	AWDM	Candida ciferrii *	MFC 50 MIC 25	Nystatin (10 µg)	[118]
	Saudi Arabia	L	H_2_O	AWDM	Candida ciferrii *	MFC 50 MIC 25	Nystatin (10 µg)	[118]
	Egypt	L	Et_2_O	GIT	Alternaria alternata *	EC_50_:167 (109–222) range	na	[69]
	Egypt	L	Et_2_O	GIT	Botrytis cinerea *	EC_50_: 462 (373–592) range	na	[69]
	Egypt	L	Et_2_O	GIT	Fusarium oxysporum *	EC_50_: 532 (413–740) range	na	[69]
	Egypt	L	Et_2_O	GIT	Fusarium solani *	EC_50_: >1000	na	[69]
	Egypt	L	Et_2_O	GIT	Alternaria alternata *	EC_50_: 229 (193–270) range	na	[69]
	Egypt	L	Et_2_O	GIT	Botrytis cinerea *	EC_50_: 245 (213–281) range	na	[69]
	Egypt	L	Et_2_O	GIT	Fusarium oxysporum *	EC_50_: 222 (182–269) range	na	[69]
	Egypt	L	Et_2_O	GIT	Fusarium solani *	EC_50_: 369 (314–453) range	na	[69]
	Iran	L	EtOH	MDM	Candida albicans *	MIC 0.25–8 range	Fluconazole	[119]
	Egypt	L, Fl	EtOH	SDB	Rhizoctonia solani *	MIC 400	Fluconazole	[120]
	Turkey	L	MeOH	BMicDM	Candida albicans *	MIC 0.39	Ampicillin (10 µg/disc), penicillin (10 µg/disc)	[121]
	Egypt	L, F	MeOH	BDM	*Aspergillus flavus* LC325160	MGI 2000	na	[122]
	Egypt	L, F	MeOH	BDM	*Cladosporium cladosporioides* *LC325159*	MGI 2000	na	[122]
	Egypt	L, F	MeOH	BDM	Penicillium chrysogenum *	MGI 2000	na	[122]
	Serbia	L	EtOH	MDM	*Alternaria alternata* DSM 2006	MIC 130.0 ± 2.9 MFC 178.0 ± 1.2	Bifonazole	[78]
	Serbia	L	EtOH	MDM	*Aspergillus flavus* ATCC 9643	MIC 178.0 ± 1.2 MFC 178.0 ± 0.6	Bifonazole	[78]
	Serbia	L	EtOH	MDM	*Aspergillus niger* ATCC 6275	MIC 178.0 ± 2.1 MFC 219.0 ± 1.5	Bifonazole	[78]
	Serbia	L	EtOH	MDM	*Aspergillus ochraceus* ATCC 12066	MIC 219.0 ± 2.3 MFC 219.0 ± 1.5	Bifonazole	[78]
	Serbia	L	EtOH	MDM	*Fusarium tricinctum* CBS 514478	MIC 178.0 ± 2.1 MFC 219.0 ± 3.5	Bifonazole	[78]
	Serbia	L	EtOH	MDM	*Penicillium ochrochloron* ATCC 9112	MIC 178.0 ± 1.2 MFC 219.0 ± 2.3	Bifonazole	[78]
	Serbia	L	EtOH	MDM	*Penicillium funiculosum* ATCC 36839	MIC 178.0 ± 0.6 MFC 219.0 ± 3.5	Bifonazole	[78]
	Serbia	L	EtOH	MDM	*Trichoderma viride* JCM 22452	MIC 267.0 ± 1.7 MFC 267.0 ± 2.0	Bifonazole	[78]
	Turkey	L	Na	BDM	*Candida parapsilosis* ATCC 22019	MIC 31.2 MFC 62.5	Fluconazole	[80]
	Turkey	L	Na	BDM	*Candida albicans* ATCC 14053	MIC > 250 MFC > 250	Fluconazole	[80]
*V. doniana*	Nigeria	L	Na	ADM	*Candida albicans MTTC* 227	IZ 36 mm	Gentamicin	[84]
*V. gardneriana*	Brazil	L	EtOH	BMicDM	*C. albicans* ATCC 90028	MIC 4	Amphotericin	[28]
	Brazil	L	EtOH	BMicDM	*C. tropicalis* LABMIC 0110	MIC 4	Amphotericin	[28]
	Brazil	L	EtOH	BMicDM	*C. parapsilosis* ATCC 22019	MIC 4	Amphotericin	[28]
	Brazil	L	EtOH	BMicDM	*C. krusei* LABMIC 0124	MIC 4	Amphotericin	[28]
*V. mollis*	Mexico	Se	MeOH	BDM	Colletotrichum gloeosporioides *	MGI 100 ± 0.0	Thiabendazole	[123]
	Mexico	Se	HX	BDM	Colletotrichum gloeosporioides *	MGI 100 ± 0.0	Thiabendazole	[123]
	Mexico	Se	EtOAc	BDM	Colletotrichum gloeosporioides *	MGI 100 ± 0.0	Thiabendazole	[123]
*V. negundo*	India	L	ButOH	AWDM	Fusarium verticillioides *	MIC 1.25	na	[124]
	India	L	MeOH	DDM	Colletotrichum gloeosporioides *	MIC 62.5	Methicillin	[91]
	Pakistan	L	MeOH	ADM	*Aspergilus niger* 0198	IZ 13.29 ± 0.72	Terbinafine	[98]
	Pakistan	L	MeOH	ADM	*Aspergilus flavus* 0064	IZ 61.06 ± 1.10	Terbinafine	[98]
	Pakistan	L	MeOH	ADM	*Aspergilus fumigates* 66	IZ 31.9 ± 0.53	Terbinafine	[98]
	Pakistan	L	MeOH	ADM	*Rhizoctonia solani* 18619	IZ 100 ± 0.00	Terbinafine	[98]
	India	L	DCM	PDB	*Alternaria alternata*	IZ 28	na	[125]
	India	L	DCM	PDB	*Cochliobolus lunatus*	IZ 14	na	[125]
	India	R	MeOH	AWDM	*Candida albicans* 3017	IZ 14.4 ± 1.6 mm	Fluconazole	[100]
	India	R	MeOH	AWDM	Candida krusei	IZ 8.9 ± 1.1 mm	Fluconazole	[100]
	India	R	MeOH	AWDM	*Candida glabrata* 3814	IZ 12.9 ± 0.8 mm	Fluconazole	[100]
	India	R	MeOH	AWDM	*Cryptococcus marinus* 1029	IZ 14.4 ± 1.6 mm	Fluconazole	[100]
	India	R	MeOH	AWDM	*Aspergillus niger* 9933	IZ 20.1 ± 1.2 mm	Fluconazole	[100]
	India	R	MeOH	AWDM	*Aspergillus brasiliensis* 1344	IZ 21.0 ± 1.0 mm	Fluconazole	[100]
	India	R	MeOH	AWDM	*Aspergillus flavus* 9607	IZ 25.9 ± 0.9 mm	Fluconazole	[100]
	India	R	MeOH	AWDM	*Rizopus oryzae*	IZ 31.5 ± 0.78 mm	Fluconazole	[100]
	India	R	MeOH	AWDM	*Epidermophyton floccosum* 7880	IZ 28.2 ± 1.2 mm	Fluconazole	[100]
	India	R	MeOH	AWDM	*Microsporum gypseum* 2819	IZ 29.8 ± 0.77 mm	Fluconazole	[100]
	India	L	EtOH	DDM	*Candida albicans* ATCC10231	IZ 00 mm	Miconazole	[101]
	India	L	HX	AWDM	*Aspergillus niger* MTCC 4325	MIC > 1000	Nystatin	[102]
	India	L	DCM	AWDM	*Aspergillus niger* MTCC 4325	MIC > 1000	Nystatin	[102]
	India	L	MeOH	AWDM	*Aspergillus niger* MTCC 4325	MIC 1000	Nystatin	[102]
	India	L	HX	AWDM	*Candida albicans* MTCC 4748	MIC 500	Nystatin	[102]
	India	L	Chl	AWDM	*Candida albicans* MTCC 4748	MIC 250	Nystatin	[102]
	India	L	MeOH	AWDM	*Candida albicans* MTCC 4748	MIC 31.2	Nystatin	[102]
	India	L	HX	AWDM	*Saccharomyces cerevisiae* MTCC 4742	MIC > 1000	Nystatin	[102]
	India	L	Chl	AWDM	*Saccharomyces cerevisiae* MTCC 4742	MIC 500	Nystatin	[102]
	India	L	MeOH	AWDM	*Saccharomyces cerevisiae* MTCC 4742	MIC 500	Nystatin	[102]
	India	L	MeOH	BMicDM	Candida albicans *	MIC 1.25	Cefotaxime	[126]
	India	B	MeOH	BMicDM	Candida albicans *	MIC 0.312	Cefotaxime	[126]
	India	L	EtOH	BMicDM	Candida albicans *	MIC 0.625	Cefotaxime	[126]
	India	B	EtOH	BMicDM	Candida albicans *	MIC 0.156	Cefotaxime	[126]
	India	L	DCM	BMicDM	Candida albicans *	MIC 2.50	Cefotaxime	[126]
	India	B	DCM	BMicDM	Candida albicans *	MIC 1.25	Cefotaxime	[126]
	India	L	PE	BMicDM	Candida albicans *	MIC 2.50	Cefotaxime	[126]
	India	B	PE	BMicDM	Candida albicans *	MIC 1.25	Cefotaxime	[126]
	India	L	MeOH	BMicDM	Candida krusei *	MIC 2.50	Cefotaxime	[126]
	India	B	MeOH	BMicDM	Candida krusei *	MIC 0.312	Cefotaxime	[126]
	India	L	EtOH	BMicDM	Candida krusei *	MIC 0.625	Cefotaxime	[126]
	India	B	EtOH	BMicDM	Candida krusei *	MIC 0.312	Cefotaxime	[126]
	India	L	DCM	BMicDM	Candida krusei *	MIC 5.00	Cefotaxime	[126]
	India	B	DCM	BMicDM	Candida krusei *	MIC 2.50	Cefotaxime	[126]
	India	L	PE	BMicDM	Candida krusei *	MIC 2.50	Cefotaxime	[126]
	India	B	PE	BMicDM	Candida krusei *	MIC 1.25	Cefotaxime	[126]
	India	L	MeOH	BMicDM	Candida parapilosis *	MIC 1.25	Cefotaxime	[126]
	India	B	MeOH	BMicDM	Candida parapilosis *	MIC 0.312	Cefotaxime	[126]
	India	L	EtOH	BMicDM	Candida parapilosis *	MIC 0.625	Cefotaxime	[126]
	India	B	EtOH	BMicDM	Candida parapilosis *	MIC 0.312	Cefotaxime	[126]
	India	L	Chl	BMicDM	Candida parapilosis *	MIC 2.50	Cefotaxime	[126]
	India	B	Chl	BMicDM	Candida parapilosis *	MIC 2.50	Cefotaxime	[126]
	India	L	PE	BMicDM	Candida parapilosis *	MIC 2.50	Cefotaxime	[126]
	India	B	PE	BMicDM	Candida parapilosis *	MIC 1.25	Cefotaxime	[126]
	India	L	MeOH	BMicDM	Candida tropicalis *	MIC 0.625	Cefotaxime	[126]
	India	B	MeOH	BMicDM	Candida tropicalis *	MIC 0.156	Cefotaxime	[126]
	India	L	EtOH	BMicDM	Candida tropicalis *	MIC 0.312	Cefotaxime	[126]
	India	B	EtOH	BMicDM	Candida tropicalis *	MIC 0.156	Cefotaxime	[126]
	India	L	DCM	BMicDM	Candida tropicalis *	MIC 2.50	Cefotaxime	[126]
	India	B	DCM	BMicDM	Candida tropicalis *	MIC 2.50	Cefotaxime	[126]
	India	L	PE	BMicDM	Candida tropicalis *	MIC 2.50	Cefotaxime	[126]
	India	B	PE	BMicDM	Candida tropicalis *	MIC 0.625	Cefotaxime	[126]
	India	L	MeOH	AWDM	Candida albicans *	IZ 5.0 ± 0.21 mm	Rifampicin	[106]
	India	L	MeOH	AWDM	Candida tropicalis *	IZ 6.1 ± 0.25 mm	Rifampicin	[106]
	India	L	MeOH	AWDM	Candida glabrata *	IZ 4.1 ± 0.29 mm	Rifampicin	[106]
	India	L	MeOH	AWDM	A. fumigatus *	IZ 3.3 ± 0.25 mm	Rifampicin	[106]
	India	L	MeOH	AWDM	A. tubingensis *	IZ 4.3 ± 0.17 mm	Rifampicin	[106]
	India	L	MeOH	AWDM	R. miehei *	IZ 4.1 ± 0.12 mm	Rifampicin	[106]
	India	L	EtOH	BMicDM	Candida albicans *	MIC 0.5	Fluconazole	[107]
	India	L	EtOH	BMicDM	Cryptococcus neoformans *	MIC 1.0	Fluconazole	[107]
	India	L	EtOH	BMicDM	Sporothrix schenckii *	MIC 2.0	Fluconazole	[107]
	India	L	EtOH	BMicDM	Trichophyton mentagrophytes *	MIC 2.0	Fluconazole	[107]
	India	L	EtOH	BMicDM	Aspergillus fumigatus *	MIC 2.0	Fluconazole	[107]
	India	L	EtOH	BMicDM	*Candida parapsilosis* ATCC-22019	MIC 1.0	Fluconazole	[107]
	India	L	EtOH	DDM	*Candida albicans* NCIM 3466	IZ 0.42 mm	Amphotericin	[108]
	India	Se	EtOH	DDM	*Candida albicans* NCIM 3466	IZ 0.52 mm	Amphotericin	[108]
	India	L	EtOH	DDM	*Trichoderma viride* NCIM 1221	IZ 0.52 mm	Amphotericin	[108]
	India	Se	EtOH	DDM	*Trichoderma viride* NCIM 1221	IZ 0.83 mm	Amphotericin	[108]
*V. trifolia*	Malaysia	L	MeOH	DDM	*Aspergillus ochraceus* NRRL 398	MIC 125	Streptomycin	[117]

ADM—Agar Dilution Method; AWDM—Agar Well Dilution Method; B—Bark; BDM—Broth Dilution Method; BMicDM—Broth Microdilution Method; ButOH—Butanol; Chl—Chloroform; DCM—Dichloromethane; DDM—Disc Diffusion Method; EC_50_—Median Inhibition Concentration; Et_2_O—Diethyl Ether; EtOH—Ethanol; F—Fruit; Fl—Flower; GIT—Growth Inhibition Technique; H_2_O—Water; HX—Hexane; IZ—Inhibition Zone; L—Leaf; MDM—Micro Dilution Method; Mean ± standard error; MeOH—Methanol; MFC—Minimum Fungicidal Concentration; MGI—Mycelial Growth Inhibition; MIC—Minimum Inhibition Concentration; na—Not available; PDB—Potato Dextrose Broth; PE—Petroliumether; R—Root; SDB—Sabouraud’s Dextrose Broth; Se—Seed; *—Strain not indicated.

**Table 4:** *In vitro* antiviral and antiprotozoal activity studies on *Vitex* species.

Species	Country	Plant Part Use	Extractive Solvent	Test Type	Strains/Microorganism	Result (µg/mL)	Positive Control	BR
*V. doniana*	Nigeria	Sb	MeOH	SDM	Plasmodium falciparum *	MIC > 4.8	Hypoxanthine	[107]
*V.* *grandifolia*	Nigeria	L	HX	PLDA	Trypanosoma brucei brucei *	IC_50_ 18.99	na	[127]
	Nigeria	L	Chl	PLDA	Trypanosoma brucei brucei *	IC_50_ 15.90	na	[127]
	Nigeria	L	MeOH	PLDA	Trypanosoma brucei brucei *	IC_50_ 8.73	na	[127]
*V. leptobotrys*	Vietnam	L	DCM	GFP	HIV-1	IC_50_ 118	Lamivudine	[128]
*V. limonifolia*	Vietnam	L	MeOH	SDM	CVB3	IC_50_ 0.12 ± 0.06	Rupintrivir	[129]
	Vietnam	L	MeOH	SDM	HRV1B	IC_50_ 48.07 ± 1.46	Ribavirin	[129]
	Vietnam	L	MeOH	SDM	EV71	IC_50_ 0.11 ± 0.05	Rupintrivir	[129]
*V. mollis*	Mexico	F	MeOH	BMicDM	Hymenolepis nana *	MIC 25	Streptomycine	[74]
*V. negundo*	India	L	EtOH	RTA	HIV-1	IZ 0.094 ± 0.01	Azidothymidine	[130]
	India	L	MeOH	BMicDM	Aedes aegypti *	IC_50_ 118.15	na	[83]
	Bangladesh	L	MeOH	BMicDM	*Agrobacterium tumefaciens* AtTa0112	IZ 6.1 ± 0.73	Camptothecin	[94]
	Bangladesh	L	MeOH	BMicDM	*Agrobacterium tumefaciens* AtAc0114	IZ 7.5 ± 0.65	Camptothecin	[94]
	Bangladesh	L	MeOH	BMicDM	*Agrobacterium tumefaciens* AtSl0105	IZ 9.3 ± 0.7	Camptothecin	[94]
*V. polygama*	Brazil	L	EtOAc	TFDM	ACVR-HSV1	MNTC 25	Rutin and Quercetin	[131]
	Brazil	L	EtOAc	TFDM	ACVR-HSV2	MNTC 26	Rutin and Quercetin	[131]
	Brazil	F	EtOAc	TFDM	ACVR-HSV1	MNTC 50	Rutin and Quercetin	[131]
	Brazil	F	EtOAc	TFDM	ACVR-HSV2	MNTC 51	Rutin and Quercetin	[131]
*V. trifolia*	India	Ap	MeOH	SDM	HSV *	MIC 2.00	na	[132]
	India	Ap	MeOH	SDM	ACV *	MIC 1.00	na	[132]

ACVR-HSV1—Acyclovir Resistant Herpes Simplex Virus Type 1; ACVR-HSV2—Acyclovir Resistant Herpes Simplex Virus Type 2; ADDM—Agar Disc Diffusion Method; Ap—Aerial part; BMicDM—Broth Micro Dilution Method; Chl—Chloroform; CVB3—coxsackievirus B3; DCM—Dichloromethane; EtOAc—Ethyl acetate; EtOH—Ethanol; EV71—Enterovirus 71; F—Flower; GFP—Green Fluorescent Protein; HIV1—Human Immunodeficiency Virus 1; HRV1B—Human rhinovirus 1B; HX—Hexane; IC_50_—Median Inhibition Concentration; IZ—Inhibition Zone; L—Leaf; IC_50_ —Inhibition Concentration; Mean ± standard error; MeOH—Methanol; MIC—Minimum Inhibition Concentration; MNTC—Maximum non-toxic Concentrations; na—Not available; PLDA—Parasite Lactate Dehydrogenase Assay; RTA—Reverse Transcriptage Assay; Sb—Steam Bark; SDM—Serial Dilution Method; TFDM—Two-fold Dilution Method; *—Strain not indicated.

**Table 5:** Isolated chemical compounds from *Vitex* species and their antimicrobial activity.

Isolated Compounds Name	Species	Microorganism	Result	BR
Agnucastoside	*V. agnus castus*	*Bacillus subtilis*	IZ 15 mm	[133]
1,8-cineole		*Micrococcus flavus*	MIC 4 µg/mL	[78]
		*Bacillus subtilis*	MIC 4 µg/mL	[78]
		*Salmonella typhimurium*	MIC 5 µg/mL	[78]
		*Staphylococcus aureus*	MIC 5 µg/mL	[78]
		*Escherichia coli*	MIC 6 µg/mL	[78]
		*Alternaria alternata*	MIC 5 µg/mL	[78]
		*Aspergillus flavus*	MIC 5 µg/mL	[78]
		*Aspergillus niger*	MIC 4 µg/mL	[78]
		*Aspergillus ochraceus*	MIC 5 µg/mL	[78]
		*Fusarium tricinctum*	MIC 3.5 µg/mL	[78]
		*Penicillium ochrochloron*	MIC 5 µg/mL	[78]
		*Penicillium funiculosum*	MIC 5 µg/mL	[78]
		*Trichoderma viride*	MIC 7 µg/mL	[78]
α-pinene		*Micrococcus flavus*	MIC 5 µg/mL	[78]
		*Bacillus subtilis*	MIC 2 µg/mL	[78]
		*Salmonella typhimurium*	MIC 8 µg/mL	[78]
		*Staphylococcus aureus*	MIC 6 µg/mL	[78]
		*Escherichia coli*	MIC 8 µg/mL	[78]
		*Alternaria alternata*	MIC 5 µg/mL	[78]
		*Aspergillus flavus*	MIC 6 µg/mL	[78]
		*Aspergillus niger*	MIC 5 µg/mL	[78]
		*Aspergillus ochraceus*	MIC 5 µg/mL	[78]
		*Fusarium tricinctum*	MIC 4 µg/mL	[78]
		*Penicillium ochrochloron*	MIC 5 µg/mL	[78]
		*Penicillium funiculosum*	MIC 6 µg/mL	[78]
		*Trichoderma viride*	MIC 8 µg/mL	[78]
Negundol	*V. negundo*	*Candida albicans*	MIC 64 µg/mL	[134]
		*Cryptococcus neoformans*	MIC 16 µg/mL	[134]
		*Trichophyton rubrum*	MIC 32 µg/mL	[134]
		*Aspergillus fumigatus*	MIC > 128 µg/mL	[134]
5-hydroxy-7, 4′ dimethoxy flavone		*Bacillus subtilis*	MIC 100 µg/mL	[135]
		*Staphylococcus aureus*	IZ 17 mm	[135]
		*Micrococcus pyogenes*	IZ 17 mm	[135]
		*Pseudomonas aeruginosa*	IZ 17 mm	[135]
		*Escherichia coli*	IZ 15 mm	[135]
5hydroxy-3,6,7,3’,4′-pentamethoxy flavone		*Bacillus subtilis*	MIC 100 µg/mL	[135]
		*Staphylococcus aureus*	IZ 18 mm	[135]
		*Micrococcus pyogenes*	IZ 17 mm	[135]
		*Pseudomonas aeruginosa*	IZ 17 mm	[135]
		*Escherichia coli*	IZ 15 mm	[135]
5,7-dihydroxy- 6,4’ dimethoxy flavanone		*Bacillus subtilis*	MIC 100 µg/mL	[135]
		*Staphylococcus aureus*	IZ 18 mm	[135]
		*Micrococcus pyogenes*	IZ 18 mm	[135]
		*Pseudomonas aeruginosa*	IZ 18 mm	[135]
		*Escherichia coli*	IZ 15 mm	[135]
5,3’-dihydroxy—7,8,4’-trimethoxy flavanone		*Bacillus subtilis*	MIC 80 µg/mL	[135]
		*Staphylococcus aureus*	IZ 18 mm	[135]
		*Micrococcus pyogenes*	IZ 17 mm	[135]
		*Pseudomonas aeruginosa*	IZ 17 mm	[135]
		*Escherichia coli*	IZ 16 mm	[135]
7,8-dimethyl herbacetin 3-rhamnoside		*Bacillus subtilis*	MIC 6.25 µg/mL	[135]
		*Staphylococcus aureus*	IZ 20 mm	[135]
		*Micrococcus pyogenes*	IZ 21 mm	[135]
		*Pseudomonas aeruginosa*	IZ 20 mm	[135]
		*Escherichia coli*	IZ 20 mm	[135]
Agnuside		*Bacillus subtilis*	MIC 12.5 µg/mL	[135]
		*Staphylococcus aureus*	IZ 19 mm	[135]
		*Micrococcus pyogenes*	IZ 20 mm	[135]
		*Pseudomonas aeruginosa*	IZ 19 mm	[135]
		*Escherichia coli*	IZ 19 mm	[135]
Negundoside		*Bacillus subtilis*	MIC 12.5 µg/mL	[135]
		*Staphylococcus aureus*	IZ 18 mm	[135]
		*Micrococcus pyogenes*	IZ 18 mm	[135]
		*Pseudomonas aeruginosa*	IZ 18 mm	[135]
		*Escherichia coli*	IZ 18 mm	[135]
Vitegnoside		*Bacillus subtilis*	MIC 6.25 µg/mL	[135]
		*Staphylococcus aureus*	IZ 20 mm	[135]
		*Micrococcus pyogenes*	IZ 21 mm	[135]
		*Pseudomonas aeruginosa*	IZ 21 mm	[135]
		*Escherichia coli*	IZ 20 mm	[135]
Vitecetin	*V. peduncularis*	*Leishmania donovani*	IC_50_ ± SD 2.4 ± 0.57	[136]

IZ—Inhibition Zone; IC_50_—Inhibition Concentration; MIC—Minimum Inhibition Concentration.

## Data Availability

The data presented in this study are openly available in Web of Science and Pubmed.
